# Synthesis, X-ray Studies and Photophysical Properties of Iridium(III) Complexes Incorporating Functionalized 2,2′:6′,2″ Terpyridines and 2,6-Bis(thiazol-2-yl)pyridines

**DOI:** 10.3390/molecules29112496

**Published:** 2024-05-24

**Authors:** Bartosz Zowiślok, Anna Świtlicka, Anna Maroń, Mariola Siwy

**Affiliations:** 1 Institute of Chemistry, University of Silesia, Szkolna 9, 40-006 Katowice, Poland; bartosz.zowislok@us.edu.pl; 2 Centre of Polymer and Carbon Materials, Polish Academy of Sciences, 34 M. Curie-Sklodowska Str., 41-819 Zabrze, Poland; msiwy@cmpw-pan.pl

**Keywords:** Ir(III) complexes, 2,2′,6,2″-terpyridine, 2,6-bis(thiazol-2-yl)pyridine, photophysical properties

## Abstract

A series of iridium(III) triimine complexes incorporating 2,2′:6′,2″-terpyridine (*terpy*) and 2,6-bis(thiazol-2-yl)pyridine (*dtpy*) derivatives were successfully designed and synthesized to investigate the impact of the peripheral rings (pyridine, thiazole) and substituents (thiophene, bithiophene, EDOT) attached to the triimine skeleton on their photophysical properties. The Ir(III) complexes were fully characterized using IR, ^1^H, elemental analysis and single crystal X-ray analysis. Their thermal properties were evaluated using TGA measurements. Photoluminescence spectra of [IrCl_3_L^1–6^] were investigated in solution at 298 and 77 K. The experimental studies were accompanied by DFT/TDDFT calculations. The photophysical properties of the synthesized triimine ligands and Ir(III) complexes were studied in detail by electronic absorption and emission. In solution, they exhibited photoluminescence quantum yields ranging from 1.27% to 5.30% depending on the chemical structure. The experimental research included DFT/TDDFT calculations. The photophysical properties of the synthesized triimine ligands and Ir(III) complexes were conducted using electronic absorption and emission techniques. In solution, they displayed photoluminescence quantum yields ranging from 1.27% to 5.30% depending on the chemical structure.

## 1. Introduction

The 2,2′:6′,2″- terpyridine (*terpy*) as a family of N–heterocyclic ligands is one of most widely studied compounds as functional templates in coordination chemistry [1]. Terpyridines were first discovered by Morgan and Burstall in 1932 during solvatothermal reaction by heating the pyridine with anhydrous FeCl_3_ [2]. Since then, numerous functional groups have been introduced into the *terpy* skeleton in different positions. The *terpy*-based metal complexes have exhibited a great potential for application in molecular magnetism, molecular electronics, catalysis, and supramolecular chemistry. It has been proven that these compounds demonstrate activity as antitumor, antimicrobial and anti-HIV agents [3,4,5,6,7]. What is more, due to their photophysical and electrochemical properties, complexes based on *terpy* derivatives have been widely studied for potential applications covering light-to-electricity conversion, light-emitting electrochemical cells (LECs), (electro)luminescent systems, non-linear optical devices, and luminescent sensors [8]. Lately, particular attention has been devoted to *terpy*-like ligands, whose structural modification consists in replacing the perihedral pyridine rings with thiazole rings leading to 2,6-bis(thiazol-2-yl)pyridines (*dtpy*). Thiazole and pyridine rings exhibit different electronic characteristics, including σ-donor and π-acceptor properties. This difference enables the tuning of HOMO and LUMO energies, thereby facilitating the development of new, efficient phosphorescent materials. In contrast to the rich coordination chemistry of 2,2′:6′,2′′-terpyridine, the number of transition metal complexes based on 2,6–(bis(thiazol-2-yl)pyridines is limited. Among polypyridine complexes of d^6^ transition metals, great attention has been devoted to iridium(III) coordination compounds, which show structural diversity and are able to form mono-, bis-, and triscyclometallated compounds [9,10,11,12,13,14,15,16,17,18]. The modification of Ir(III) environment in those three types of compounds allows for tuning their emission properties that cover the full visible spectra up to the near infrared. The high structural variety allows better control of the excited state nature and emission properties of these complexes and Ir(III) species are known to exhibit higher triplet quantum yields due to the mixing of the singlet and triplet excited states through spin-orbital coupling, leading to high phosphorescence efficiencies [17,19].

**Scheme 1:**
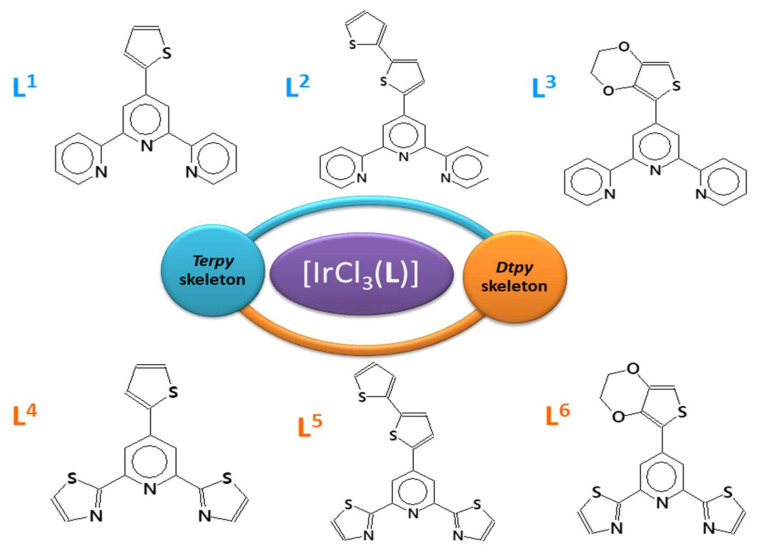
Ligands used in the synthesis of the complexes.

The main aim of this work was to explore the impact of the triimine skeleton (*terpy* and *dtpy*) and substituents (thiophene, bithiophene, and EDOT) on the photophysical properties of Ir(III) complexes. Recently, some efforts have been made to understand the structure–property correlation in rhenium(I), platinum(I), gold(I), cobalt(II), and copper(II) [20,21,22,23,24,25,26] complexes by introducing the R substituent in the 4′-position of the *terpy* and/or *dtpy* rings. However, to the best of our knowledge, [IrCl_3_(L)] complexes incorporating *terpy* (ligands L^1^–L^3^*)* and *dtpy* derivatives (ligands L^4^–L^6^) are quite rare and will be reported here for the first time (see Table S15). Taking into account the ability to tune the luminescent properties of [IrCl_3_(4′-R-terpy)] and [IrCl_3_(4′-R-dtpy)], we chose Ir(III) complexes in order to investigate their photophysical properties. The used R substituents (Scheme 1) incorporated into the 4′-position of the *terpy*/*dtpy* skeleton have different electron donating or withdrawing properties as well as different steric effects. The luminescent properties were studied in solution at 298 K and 77 K. The density functional theory and time-dependent DFT calculations were performed to obtain insight into their electronic structure and spectroscopic properties.

## 2. Results and Discussion

### 2.1. Synthesis and Structural Characterization

To synthesize iridium(III) complexes, the procedure based on the solvatothermal reaction of IrCl_3_ with an appropriate N-donor ligand in 2-methoxyethanol was employed. The successful coordination of the triimine ligands and formation of Ir(III) complexes have been confirmed by elemental analysis and spectroscopic tools (Figures S1 and S2) and X-ray diffraction studies. The last ones were performed for monocrystals of **1** and **6**.

**Figure 1:**
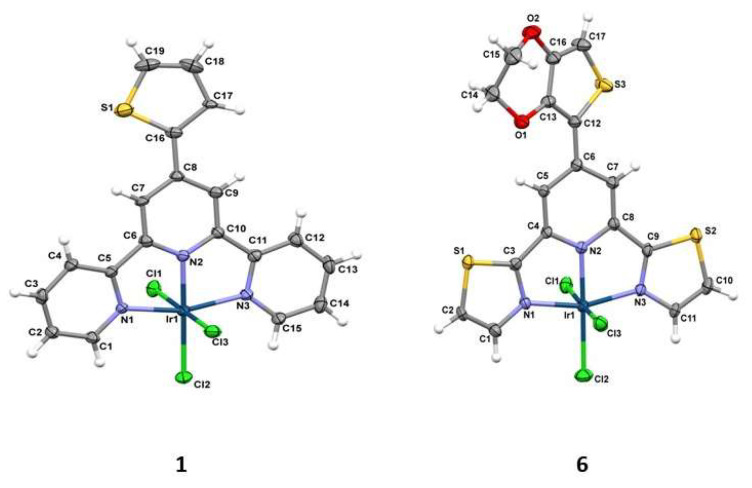
Molecular structures of the compounds **1** and **6** together with the atom numbering. Displacement ellipsoids are drawn at the 50% probability level.

The crystallographic studies together with selected bond distances and angles are summarized in Tables S1 and S2 in the Supporting Information. The molecular structures of **1** and **6** as representative examples are depicted in Figure 1. The coordination of tridentate *terpy/dtpy* ligands results in significant distortions within the typically octahedral environment surrounding the iridium atom. Whereas the bond angles of Cl(1)–Ir(1)–Cl(3) (178.82(5)° in **1**; 177.32(6)° in **6**) and N(2)–Ir(1)–Cl(2) (177.30(16)° in **1**; 177.75(14)° in **6**) are both close to the ideal angle of 180°, that of N(1)–Ir(1)–N(3) is much smaller (161.00(17)° in **1**; 160.4(2)° in **6**) owing to the chelating coordination mode of modified ligand formation of two five-membered metallocycles. Notably, [IrCl_3_(L)] complexes (L = tridentate N-donor ligand) are relatively rare [27,28,29,30,31,32,33]. A search in the Cambridge Structure Database (Version 5.37) revealed only nine such systems (Table S10).

**Figure 2:**
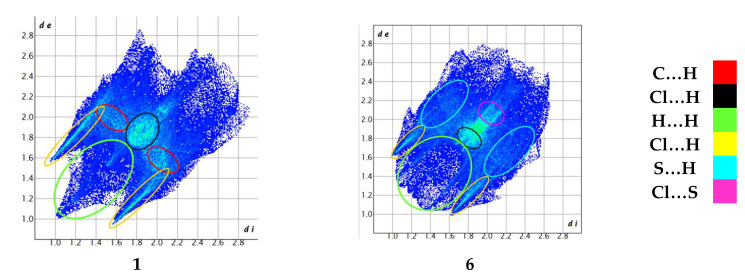

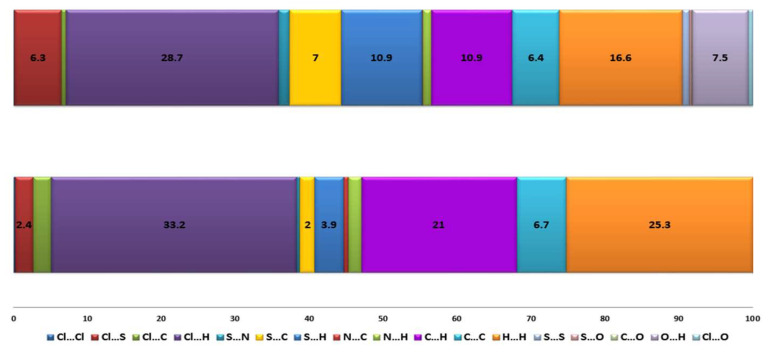
Hirshfeld surface mapped with d_norm_ (**top**), graph showing a fraction of the individual close contacts on the Hirshfeld surfaces (**down**).

The central bond length Ir(1)–N(2) (1.942(4)Å in **1**; 1.960(5)Å in **6**) is significantly shorter than other two Ir(1)–N bond distances; this leads to a lengthening of the trans sited Ir(1)–Cl(2) bond distances. The crystal packing analysis (Mercury 3.10.2 program) [34] demonstrates that the molecules of **1** and **6** show weak C–H⋯S/π⋯π and C–H⋯O/C–H⋯S/C–H⋯Cl/π⋯π interactions, respectively. More structural details are given in Tables S3 and S4. The Hirshfeld surfaces were generated using Crystal Explorer 21.5 in order to quantify and visualize the intermolecular interactions in the crystal structures (Figure 2) [35,36].

The Hirschfeld surfaces confirm that the C-H…Cl together with van der Waals interactions play a dominant role in the examined structures, the C…H, Cl…H, and H…H contributions are significant (21%, 33.2%, and 25.3% in **1** and 10.9%, 28.7%, and 16.6% in **6**). In **6**, the S…C (7%) and S…H (10.9%) are important in confirming the results of the intermolecular interactions analysis (see Tables S3 and S4 in ESI). In **6**, the C(5)–H(5)…O(3) and C(11)–H(11)…O(2) interactions seems to be among the most important, and the O…H contribution (7.5%) confirms that the 3,4-ethylenedioxythiophene substituent in the *dtpy* skeleton actively participates in the creation of the interaction network.

### 2.2. Thermal Properties

In order to examine the thermal properties of [IrCl_3_(L^1^–L^6^)] complexes, thermogravimetric analysis (TGA) was applied. The results are collected in Table S5 and Figure S6. The TGA results show that the Ir(III) complexes based on the *terpy* skeleton are more stable than those based on *dtpy* derivatives; however, the effect of the substituent is also significant. The values of T_max_ of the Ir(III) compounds EDOT-terpy (**3**) (423°) and EDOT-dtpy (**6**) (438°) are less stable than thiophene (508° in **1**; 450° in **4**) and bithiophene (493° in **2** and 466° in **5**) derivatives. At these temperatures, the TG curves display one weight loss attributed to the release of the R substituent: 14.142% in **1** (calcd 13.54%); 22.218% in **3** (calcd 22.49%); 12.692% in **4** (calcd 13.26%); 23.30% in **5** (calcd 21.073%); 21.164% in **6** (calcd 20.61%). The compound **2** exhibits different thermal behavior than the other compounds due to the two-step decomposition of the bithiophene substituent. The first weight loss is 13.362% at 525° (calcd 23.71%), which can be assigned to the release of only one thiophene ring. Further bithiophene decomposition occurs at a higher temperature and seems to be accompanied by the decomposition of the *terpy* skeleton.

### 2.3. Electronic Absorption Spectra and TD-DFT Calculations

**Figure 3:**
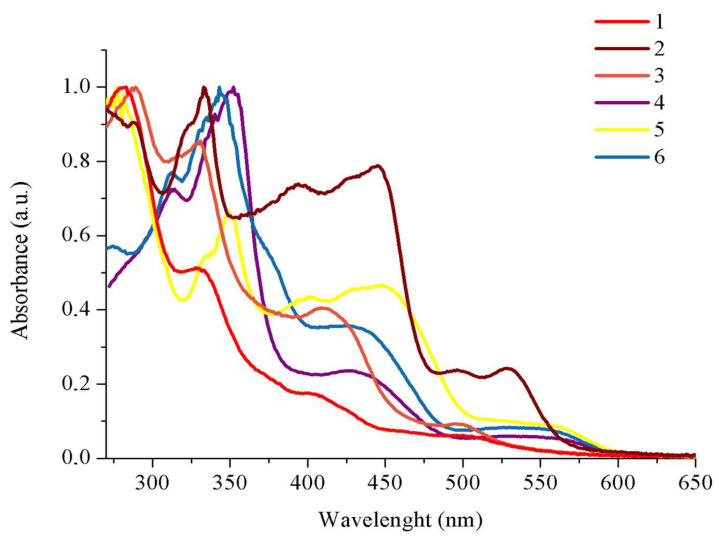
UV–Vis profiles of **1**–**6** recorded in DMSO.

The electronic absorption spectra of **1**–**6** registered in DMSO are presented in Figure 3, and the relevant spectroscopic data are summarized in ESI. Comparing the spectra of the free ligands L^1^–L^6^, the high energy absorptions of **1**–**6** can be assigned to the π → π* transitions localized on the triimine ligand coordinated to the metal center in a tridentate mode (see Figure S4). The absorptions of **1**–**6** in the low-energy region with maxima in the range 498–563 nm and much lower molar extinction coefficients possess a charge transfer character. The maxima of the complexes **4**–**6** based on the *dtpy* skeleton are significantly red-shifted (550 nm in **4**, 563 nm in **5**, 535 in **6**) in comparison to the compounds **1**–**3** incorporating ligands with the *terpy* core (508 nm in **1**, 530 nm in **2**, 498 nm in **3**). Considering the influence of the R substituents, it can be noticed that the bithiophene Ir(III) derivatives (**2** and **5**) are the most red-shifted in comparison to the thiophene (**1** and **4**) and EDOT (**3** and **6**) Ir(III) systems (see Table S6).

**Figure 4:**
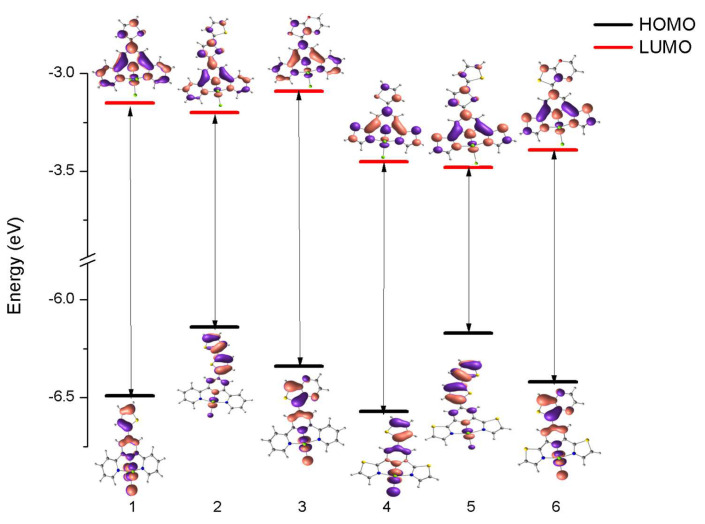
Molecular orbital energy level graph of **1**–**6** at the DFT/B3LYP/LAN2DZ/6-31G* level.

To deepen our understanding of the nature of the excited states involved in absorption processes, DFT/TD-DFT calculations were conducted for **1**–**6**. The optimized structures of **1** and **6** agreed with the X-ray data (Table S2 in the ESI). An analysis of the molecular orbital energy levels graph for **1**–**6** reveals that energy gaps persist within the range of 2.69–3.33 eV. (Figure 4) The HOMO–LUMO gap is significantly lower in **2** (2.93 eV) and **5** (2.69 eV), which confirmed that the optical profile of [IrCl_3_(4′-bithiophene-*terpy/dtpy*)] differs from the other studied compounds. Moreover, the LUMO levels of **4**–**6** are destabilized in comparison with those for **1**–**3**, which indicates significant differences between the *terpy* and *dtpy* core of the tridentate ligand. The HOMO orbitals for the pairs of the Ir(III) complexes incorporating thiophene and EDOT as R substituents lie in similar energy levels: –6.49 eV; –6.57 for **1**/**4** and –6.34 eV; –6.42eV for **3**/**6**, respectively.

**Figure 5:**
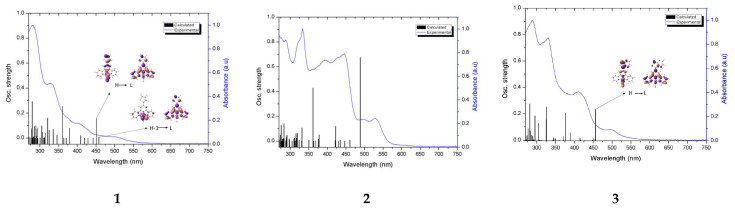

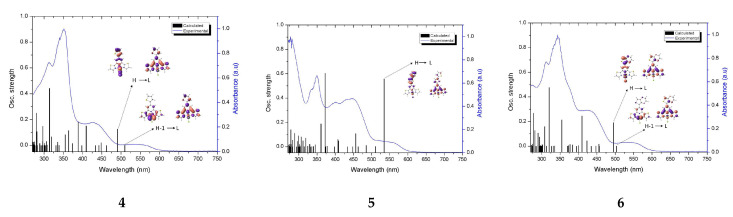
Experimental (blue line) absorption spectra of **1**–**6** in DMSO alongside transitions (black lines) computed at the DFT/B3LYP/LAN2DZ/6-31G* level.

The experimental and calculated electronic absorption spectra of **1**–**6** are compared in Figure 5. According to the theoretical calculations, the low-energy absorption band is composed of several transitions of different origins (see Tables S7–S12). Predominant contributions can be assigned to the excitations H-1→LUMO; HOMO→LUMO in **1**, **4** and **6**; and HOMO→LUMO in **2**, **3**, and **5**. Based on the MO composition (Figure S5, Table S13) in **1**, **3**, and **6**, the transitions H–1→LUMO can be assigned to MLCT [dπ(Ir)→π*(triimine)], while HOMO→LUMO possess an admixture of ILCT transition (charge transfer with electronic density originating from the R substituent unit to the π-conjugated triimine acceptor moiety. As previously described, the complexes **2** and **5** differ from the other complexes and their HOMO→LUMO transitions are dominated by intense ILCT transition (πR/→ π*(terpy) in **2**; πR/→ π*(dtpy) in **5**).

### 2.4. Emission Properties

**Figure 6:**
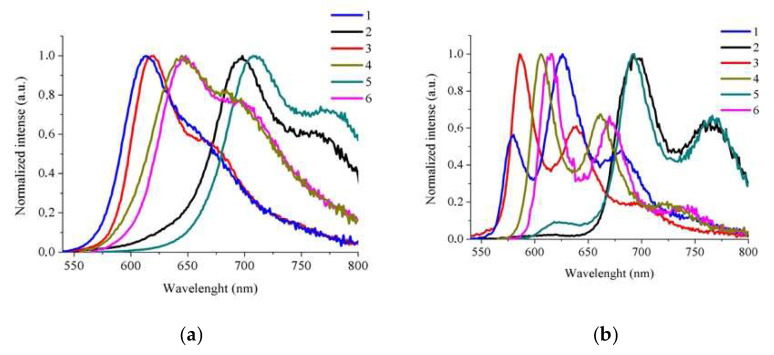
The normalized emission spectra of **1**–**6** in DMSO at room temperature (**a**) and in a low-temperature matrix (77 K in EtOH–MeOH (4:1 *v*/*v*)) (**b**).

The photoluminescence properties of [IrCl_3_(L^1^–L^6^)] (**1**–**6**) were investigated in deaerated DMSO solution at room temperature and in the EtOH–MeOH (4:1 *v*/*v*) rigid matrix at 77 K. All the Ir(III) complexes were found to be emissive under these experimental conditions and the summary of the photophysical data is presented in Table 1 and Figure 6.

The excitation of iridium(III) complexes at the lowest absorption band gave rise to a room-temperature structured emission band with a maximum located in the orange-red spectral region (612–709 nm). The emission behavior of **1**–**6** is sensitive to the donor–acceptor properties of substituents incorporated into *terpy* (**1**–**3**) and *dtpy* skeletons (**4**–**6**). The attachment of thiazole and EDOT groups to the *terpy* ligand in the complexes **1** and **3** provides a broad band with weak shoulder with maxima at 612 nm, 660 nm (sh) nm in **1**; and 620 nm, 677 nm (sh) nm in **3**. The same effect is observed for the pair of compounds based on the *dtpy* skeleton. The bands of **4** and **6** showed a more distinct shoulder with red-shifted maxima by ca. 30 nm. The compounds **2** and **5** containing bithiophene as a substituent are the most shifted with two well-separated bands towards red wavelengths with the maxima 698 nm, 767 nm for **2** and 709 nm, 771 nm for **5**. The emission lifetimes fall in the microsecond time regime in the case of the complexes **1**, **2**, and **5**, while the compounds **3**, **4**, and **6** have nanosecond lifetimes. The decay curves for all of the Ir(III) complexes described by a biexponential fit indicate emission from more than one excited state (^3^MLCT mixed with ^3^IL/^3^ILCT). The luminescence quantum yields for Ir(III) based on the *terpy* skeleton are higher (4.42–5.30%) in comparison to *dtpy*–Ir(III) derivatives.

At low temperature (77 K), strong vibronic coupling features emerge, clearly indicating that the ligand affects the lowest-energy excited state (^3^IL/^3^ILCT).

The emission slightly shifts to higher energies and shows significant increases in the excited state lifetimes relative to room temperature.

### 2.5. Electrochemistry

The electrochemical properties of the Ir(III) complexes were studied at room temperature in DMF. The obtained data are gathered in Table S14 in ESI. No oxidation processes were observed for 1–6 up to +2.00 V. In general, it is expected that metal-based oxidation is shifted towards more positive potentials; so in terpy–like Ir(III) systems, Ir^IV^/Ir^III^ redox couple cannot be observed in the conventional potential window [37]. For **1**–**6**, one reversible one-electron reduction process can be observed from −1.63 to −1.43V. By comparison to similar Ir(III) complexes, this process can be attributed to the reduction of the terpyridine/2,6-bis(thiazol-2-yl)pyridine ligands, and the LUMO may be located on the π* orbital of the polypyridyl ligand.

## 3. Materials and Methods

**Scheme 2:**
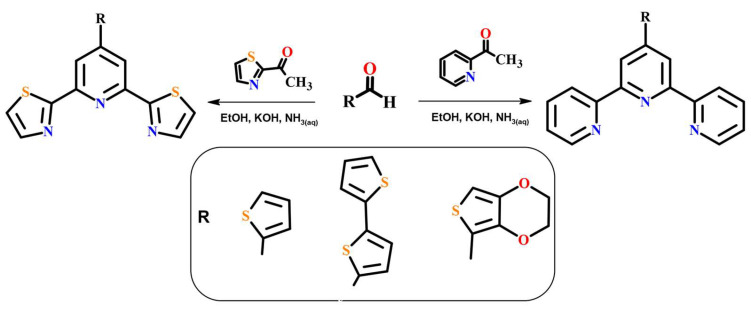
Ligands L^1^–L^6^: Synthetic route.

As a starting materials, IrCl_3_ was employed, while solvents of reagent grade were utilized for ligand synthesis. The solvents (HPLC grade) were used for spectroscopic studies. All materials were commercially available and used without additional purification. The N–heterocyclic ligands (L^1^–L^5^) were synthesized following literature procedures involving the condensation of 2-acetylpyridine or 2-acetylpyridine with appropriate commercially available benzaldehyde [38,39,40]. The 3,4-(Ethylenedioxy)thiophene-2-carboxaldehyde for ligand L^6^ was prepared according to the literature method [41] (Scheme 2).

Data were processed with the aid of CrysAlis Pro software [42], Olex2 software [43], SHELXT, and SHELXL-2018/3 package [44]. Single crystals of **1** and **6** were grown from acetonitrile by slow evaporation of the solvent at room temperature. X-ray diffraction was performed using an Oxford Diffraction four-circle diffractometer Gemini A Ultra model, equipped with an Atlas CCD detector. Graphite-monochromated MoK radiation with a wavelength (λ) of 0.71073 Å was utilized for data collection, conducted at room temperature. CrysAlis Pro software [42], Olex2 software [43], SHELXT, and the SHELXL-2018/3 package [44] were used for data processing. All the non-hydrogen atoms were refined anisotropically, and hydrogen atoms were placed in calculated positions and refined with riding constraints: d(C–H) = 0.93 Å, Uiso(H) = 1.2 Ueq(C) (for aromatic) and d(C–H) = 0.96 Å, Uiso(H) = 1.5 Ueq(C) (for methyl). Crystallographic data for **1** and **6** were deposited with the Cambridge Crystallographic Data Center, CCDC 2352214-2352215.

IR spectra were obtained using a Nicolet iS5 FT-IR spectrophotometer (ThermoScientific, Waltham, MA, USA) within the range of 4000–400 cm^−1^. Electronic absorption spectra were recorded in DMSO with a concentration of c = 2.5 × 10^−5^ mol dm^−3^ using a Nicolet Evolution UV-VIS-NIR spectrophotometer (range: 650–250 nm). The ^1^H NMR spectra for **1**–**6** were produced at room temperature in DMSO-d^6^ using a Bruker 400 MHz spectrometer, while elemental analyses (C, H, N) were carried out on a Perkin–Elmer CHN-2400 analyzer (Perkin–Elmer, Waltham, MA, USA). The FLS-980 spectrofluorimeter (Edinburgh Instruments Ltd., Livingston, Scotland) were used for recording photoluminescence spectra. The room-temperature spectra were prepared in DMSO (c = 2.5 × 10^−5^ mol dm^−3^). The low-temperature emission spectra were measured in an ethanol:methanol mixture (4:1) frozen-glass matrix at the temperature of liquid nitrogen with a Dewar assembly. The time-resolved measurements were conducted at optically diluted solutions with an optical density ranging from 0.05 to 0.1 at room temperature. The experiments utilized time-correlated single photon counting (TCSPC) methods on the FLS-980 spectrofluorimeter. The excitation wavelength was obtained using a set of picosecond pulsed diodes, specifically EPLED–340 nm and EPLED–375 nm, serving as the light source. A PMT (Hamamatsu, R928P) housed in a cooled environment was employed as the detector, with the system alignment performed at the emission wavelength. Additionally, to analyze fluorescence decay, an instrument response function (IRF) was acquired. This IRF provides details regarding the time response of the entire optical and electronic system. For its determination, a ludox solution was utilized as a standard at the excitation wavelengths. Quantum yields were measured using the integrating sphere absolute method at room temperature and using the solvent (DMSO) as a blank. The samples were optically diluted to avoid the inner filter effect and re-absorption.

Cyclic voltammetry (CV) and differential pulse voltammetry (DPV) measurements were performed using the Autolab potentiostat (Eco Chemie) under ambient conditions. A three-electrode, one-compartment cell was utilized to contain the deaerated solution of complexes and supporting electrolyte in DMF. The complexes and supporting electrolyte (n-Bu4NPF6) concentrations were equal to 10^−6^ mol dm^−3^ and 0.01 mol dm^−3^, respectively. The scan rate was equal to 0.1 V s^−1^. A glassy carbon disk working electrode (3 mm diam.) and a Ag/Ag+ reference electrode were used. TGA for **1**–**6** was carried out on a Perkin Elmer Pyris1 instrument (Perkin Elmer, Waltham, MA, USA).

### Preparation of Iridium(III) Complexes

IrCl_3_ (1 mmol) and appropriate ligands L^1^–L^6^ (1 mmol) were dissolved in 2-methoxyethanol (25 mL). The resulting solution was heated in an autoclave for 20 h to 150 °C, maintained at this temperature for 30 h, and then cooled down to room temperature for another 30 h. X-ray-quality brown (**1**, **6**) crystals were obtained by filtration and were washed with diethyl ether.

**1**: Yield: 60%. Elemental analysis calcd: C 37.17%, H 2.13%, N 6.84% found: C 37.22%, H 2.18%, N 7.00%; ^1^H NMR (500 MHz, DMSO) δ 9.22 (ddd, *J* = 5.6, 1.4, 0.5 Hz, 2H), 9.00 (s, 2H), 8.92 (d, *J* = 8.0 Hz, 2H), 8.31 (td, *J* = 7.8, 1.5 Hz, 2H), 8.26 (dd, *J* = 3.7, 1.1 Hz, 1H), 8.00–7.96 (m, 3H), 7.42 (dd, *J* = 5.0, 3.7 Hz, 1H) Ir (KBR; cm^–1^): 1604 (vs), 1424 (vs), 1254 (m), 786 (vs), 724 (s).

**2**: Yield: 65%. Elemental analysis calcd: C 39.69%, H 2.17%, N 6.04% found: C 39.58%, H 2.27% N, 6.37%; ^1^H NMR (500 MHz, DMSO) δ 9.22 (d, *J* = 7.1 Hz, 2H), 9.00 (s, 1H), 8.93 (d, *J* = 7.6 Hz, 2H), 8.31 (dd, *J* = 16.9, 2.8 Hz, 3H), 8.26 (d, *J* = 4.0 Hz, 1H), 8.02–7.97 (m, 2H), 7.69–7.66 (m, 2H), 7.54 (d, *J* = 3.6 Hz, 1H), 7.22 (dd, *J* = 5.1, 3.6 Hz, 1H). Ir (KBR; cm^–1^): 1606 (vs), 1464 (m) 1445 (vs), 1242 (m), 788 (s).

**3**: Yield: 70%. Elemental analysis calcd: C 37.42%, H 2.54%, N 6.23%; found C 37.67%, H 2.45%, N 6.56%; ^1^H NMR (500 MHz, DMSO) δ 9.22 (d, 2H), 8.81–8.74 (m, 4H), 8.27 (t, 2H), 7.98 (t, 2H), 7.09 (s, 1H), 4.54 (s, 2H), 4.38 (s, 2H). Ir (KBR; cm^–1^): 1604 (s), 1495 (vs), 1474 (s), 1420 (m), 1358 (m), 1060 (m), 790 (m)

**4**: Yield: 60%. Elemental analysis calcd: C 28.78%, H 1.45%, N 6.71%; found: C 28.46%, H 1.43%, N 6.77%; ^1^H NMR (500 MHz, DMSO) δ 8.94 (s, 2H), 8.59 (d, *J* = 3.4 Hz, 2H), 8.27 (d, *J* = 3.4 Hz, 2H), 8.23 (d, *J* = 3.7 Hz, 1H), 7.95 (d, *J* = 6.0 Hz, 1H), 7.41–7.38 (m, 1H). Ir (KBR; cm^–1^): 1606 (vs), 1482 (m), 1247 (m), 740 (m), 726 (w).

**5**: Yield: 60%. Elemental analysis calcd: C 32.23%, H 1.57%, N 5.93%; found: C 31.96%, H 1.84%, N 6.01%; ^1^H NMR (500 MHz, DMSO) δ 8.96 (s, 2H), 8.59 (d, *J* = 3.3 Hz, 2H), 8.27 (d, *J* = 3.4 Hz, 2H), 8.23 (d, J = 4.0 Hz, 1H), 7.68 (dd, *J* = 5.1, 1.1 Hz, 1H), 7.63 (d, *J* = 3.9 Hz, 1H), 7.55 (dd, *J* = 3.6, 1.1 Hz, 1H), 7.22–7.21 (m, 1H). Ir (KBR; cm^–1^): 1601 (vs), 1464 (vs), 1246 (m), 732(m).

**6**: Yield: 70%. Elemental analysis calcd: C 29.68%, H 1.77% N 6.13%; found: C 30.68%, H 1.85% N 6.45%; ^1^H NMR (500 MHz, DMSO) δ 8.68 (s, 2H), 8.59 (d, *J* = 3.4 Hz, 2H), 8.27 (d, *J* = 3.4 Hz, 2H), 7.09 (s, 1H), 4.55 (s, 4H), 4.38 (s, 4H). Ir (KBR; cm^–1^): 1599 (s), 1495 (vs), 1436 (v), 1358 (w), 1177 (m), 1059 (s), 763 (m).

## 4. Conclusions

Concluding, we present the synthesis and structural, thermal, electrochemical, and photophysical characterization of iridium(III) complexes of the general formula [IrCl_3_(L^1−6^)]. The adaptation of synthesis method to solvatothermal conditions gave rise to synthesized complexes with good yields (60–70%) and of the high purity, which was confirmed via FT-IR, NMR, and elemental analysis. Additionally, for the complexes **1** and **6**, the X-ray analysis has been performed, confirming a pseudooctahedral structure around iridium(III) ions. Crystal packing analysis together with Hirshfeld surface analysis indicates 3D supramolecular assembly in both complexes. The compounds **1**–**6** display good thermal stability with T_max_ values in the range 450–508 °C depending on the triimine skeleton and substituent attached. CVs and DPVs show only ligand-based reduction without an oxidation wave, indicating that the LUMOs have a π*_(terpy/dtpy)_ character. The HOMOs, estimated via DFT, are in the majority localized on substituents. The lowest-energy absorption band is sensitive to both the type of triimine skeleton as well as the kind of substituent attached. Accordingly to TD-DFT calculations, this band can be attributed to MLCT/IL/ILCT transitions for **1**, **3** and **4**, **6**, while it became predominately ILCT in the case of **2** and **5**. The complexes display room-temperature red-orange phosphorescence in solution with quantum yields in the range 1.27–5.30% and lifetimes of hundreds of nanoseconds to a few microseconds. The structured shape of the emission band at both room and low temperatures indicates a substantial share of the ligand in the emission process. The emission bands of bithiophene-substituted compounds (shifted to the red) may be associated with ^3^ILCT contribution, while in the case of thiophene- and EDOT-appended based complexes the emitting state is more mixed between ^3^MLCT and ^3^IL/^3^ILCT. Our studies clearly evidence that the substituent attached to the triimine skeleton governs the character of LEES, while the triimine skeleton plays a vital role in the overall ratio between the radiative and non-radiative processes influencing the quantum yield and lifetime of the excited state. We also plan to examine early photophysical processes for [Ir(L)(terpy)]/[Ir(L)(dtpy)], where L represents N-donor polypyridine and/or cyclometalating ligands. Among the six Ir(III) compounds, the compounds **2** and **5** seem to be the most interesting and will be used for the preparation of bis(terpy)/bis(dtpy) complexes of iridium(III). The compounds **2** and **5** definitely differ from the other Ir(III) complexes, taking into account the nature of their HOMO→LUMO transitions as well as their photophysical profiles.

## Figures and Tables

**Table 1:** Photoluminescent data (excitation λ_ex_ and emission λ_em_ wavelength, quantum yield Φ, lifetime τ, and goodness-of-fit-χ^2^) of **1**–**6** in DMSO solution (5 × 10^−5^M), as well as in solid state and 77 K glass (EtOH-MeOH,4:1, *v*/*v*).

Compound	Medium	λ_em_ [nm]	λ_ex_ [nm]	ϴ [%]	τ [ns]	χ^2^
**1**	DMSO	612, 660 (sh)	525	4.69	866 (24%) 4337 (76%)	1.091
	77 K	579, 625, 681	505		1760 (15%), 11,630 (65%)	1.134
					38 880 (20%)	
**2**	DMSO	698, 767	530	5.30	4914.5 (96%) 17115.5 (4%)	1.018
	77 K	693, 766	523		12745	0.952
**3**	DMSO	620, 677 (sh)	525	4.42	1899.5 (46%) 6603 (54%)	1.091
	77 K	586, 638	496		6450 (17%) 14,894 (83%)	1.002
**4**	DMSO	642, 683 (sh)	535	4.72	202 (84%), 1257 (16%)	1.091
	77 K	605, 661	520		15437	1.013
**5**	DMSO	709, 771	540	1.62	5734 (62%) 12,812 (38%)	0.961
	77 K	691, 766	545		5300 (4%) 15,617 (96%)	0.993
**6**	DMSO	648, 705 (sh)	535	1.27	206 (79%) 1197 (21%)	1.108
	77 K	616, 670	529		59 (7.5%) 7728 (14.5%) 14,939 (78%)	1.056

## Data Availability

Data are contained within the article and Supplementary Materials.

## Supplementary Materials

The following supporting information can be downloaded at: https://www.mdpi.com/article/10.3390/molecules29112496/s1. Refs [45,46] are cited in the Supplementary Materials. Figure S1. FT-IR spectra of **1**–**6**; Figure S2. ^1^H NMR spectra of **1**–**6** (a) and ^1^H NMR and ^13^C NMR spectra of L^6^ (b); Figure S3. TGA of **1–6** under dry N_2_ atmosphere; Figure S4. UV-Vis spectra of complexes **1–6** together with ligand L^1^–L^6^; Figure S5. Composition of frontier molecular orbitals of complexes **1–6** (blue – Ir, red – 3Cl green – R’ substituent, violet – *terpy*/*dtpy* skeleton). Table S1. Crystal data and structure refinement; Table S2. Selected bond lengths (Å) and angles (deg) for **1** and **6**; Table S3. Short intra–and intermolecular contacts; Table S4. Short π•••π stacking interactions; Table S5. TGA data for **1**–**6**; Table S6. The absorption maxima for complexes [IrCl_3_(L^1^–L^6^)] (**1–6**); Table S7. The energies and characters of the selected spin-allowed electronic transitions for **1** calculated with the TDDFT/PBE1PBE method, together with assignment to the experimental absorption bands; Table S8. The energies and characters of the selected spin-allowed electronic transitions for **2** calculated with the TDDFT/PBE1PBE method, together with assignment to the experimental absorption bands; Table S9. The energies and characters of the selected spin-allowed electronic transitions for **3** calculated with the TDDFT/PBE1PBE method, together with assignment to the experimental absorption bands; Table S10. The energies and characters of the selected spin-allowed electronic transitions for **4** calculated with the TDDFT/PBE1PBE method, together with assignment to the experimental absorption bands; Table S11. The energies and characters of the selected spin-allowed electronic transitions for **5** calculated with the TDDFT/PBE1PBE method, together with assignment to the experimental absorption bands; Table S12. The energies and characters of the selected spin-allowed electronic transitions for **6** calculated with the TDDFT/PBE1PBE method, together with assignment to the experimental absorption bands; Table S13. Frontier molecular orbitals of complexes **1–6**; Table S14. Electrochemical properties of the Ir(III) complexes in DMF; Table S15. Structural and photophysical comparison of related Ir(III) complexes.
