# Supplementary material for: Synthesis, X-ray Studies and Photophysical Properties of Iridium(III) Complexes Incorporating Functionalized 2,2′:6′,2″ Terpyridines and 2,6-Bis(thiazol-2-yl)pyridines
Source: Molecules. 2024 May 24;29(11):2496. doi: 10.3390/molecules29112496 (PMC11173833; doi:10.3390/molecules29112496)
Supplement: Supplementary file 1 [file molecules-29-02496-s001.zip › molecules-3021667-supplementary.pdf]

Electronic Supplementary Information

for

Synthesis, X-ray studies and photophysical properties of iridium(III) complexes incorporating functionalized 2,2':6',2" terpyridines and 2,6-bis(thiazol-2-yl)pyridines

Bartosz Zowiślok[1], Anna Świtlicka [1*], Anna Maroń [1*] and Mariola Siwy [2]

[1.] Institute of Chemistry, University of Silesia, Szkolna 9, 40-006 Katowice, Poland
[2.] Centre of Polymer and Carbon Materials, Polish Academy of Sciences, 34 M. Curie-Sklodowska Str., 41-819 Zabrze, Poland

General characterization 3
Figure S1. FT-IR spectra of **1-6**. 4
Figure S2. ^{1}H NMR spectra of **1-6** 8
X-Ray studies 8
Table S1. Crystal data and structure refinement 8
Table S2. Selected bond lengths (Å) and angles (deg) for **1** and **6**. 9
Table S3. Short intra–and intermolecular contacts 10
Table S4. Short π•••π stacking interactions 10
Thermal properties 11
Table S5. TGA data for **1**–**6** 11
Figure S3. TGA of **1-6** under dry N_2 atmosphere 11
Spectroscopic studies 11
Table S6. The absorption maxima for complexes [IrCl3(L^1–L^6)] (**1-6**) 11
Figure S4. UV-Vis spectra of complexes **1-6** together with ligand L^1–L^6 12

Table S7. The energies and characters of the selected spin-allowed electronic transitions for **1** calculated with the TDDFT/PBE1PBE method, together with assignment to the experimental absorption bands. 12

Table S8. The energies and characters of the selected spin-allowed electronic transitions for **2** calculated with the TDDFT/PBE1PBE method, together with assignment to the experimental absorption bands. 13

Table S9. The energies and characters of the selected spin-allowed electronic transitions for **3** calculated with the TDDFT/PBE1PBE method, together with assignment to the experimental absorption bands. 13

Table S10. The energies and characters of the selected spin-allowed electronic transitions for **4** calculated with the TDDFT/PBE1PBE method, together with assignment to the experimental absorption bands. 14

Table S11. The energies and characters of the selected spin-allowed electronic transitions for **5** calculated with the TDDFT/PBE1PBE method, together with assignment to the experimental absorption bands. 14

Table S12. The energies and characters of the selected spin-allowed electronic transitions for **6** calculated with the TDDFT/PBE1PBE method, together with assignment to the experimental absorption bands. 15

Figure S5. Composition of frontier molecular orbitals of complexes **1-6** (blue – Ir, red – 3Cl green – R' substituent, violet – *terpy*/*dtpy* skeleton). 17

Table S13. Frontier molecular orbitals of complexes **1-6**. 18

Electrochemistry 20

Table S14. Electrochemical properties of the Ir(III) complexes in DMF. 20

General characterization

1

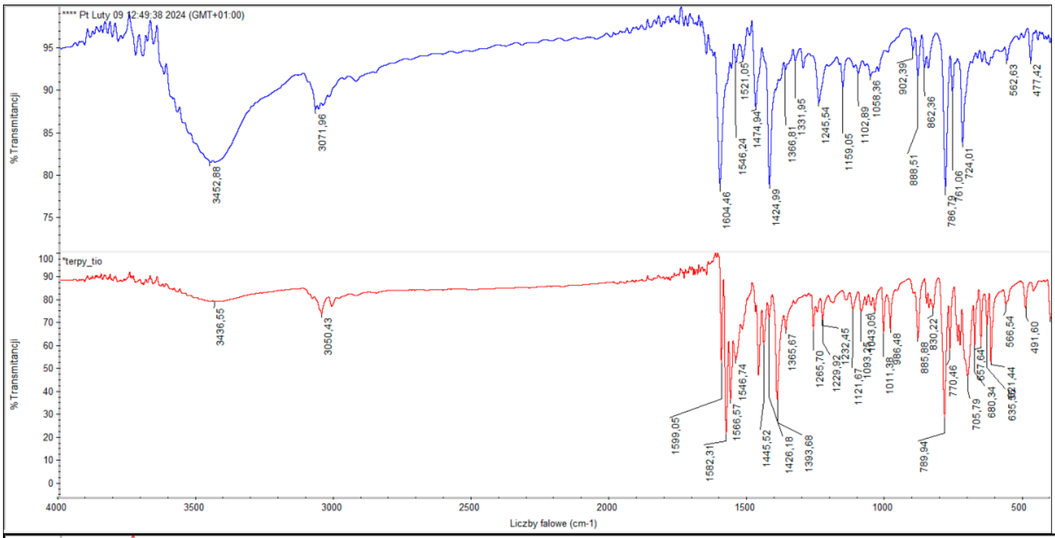

2

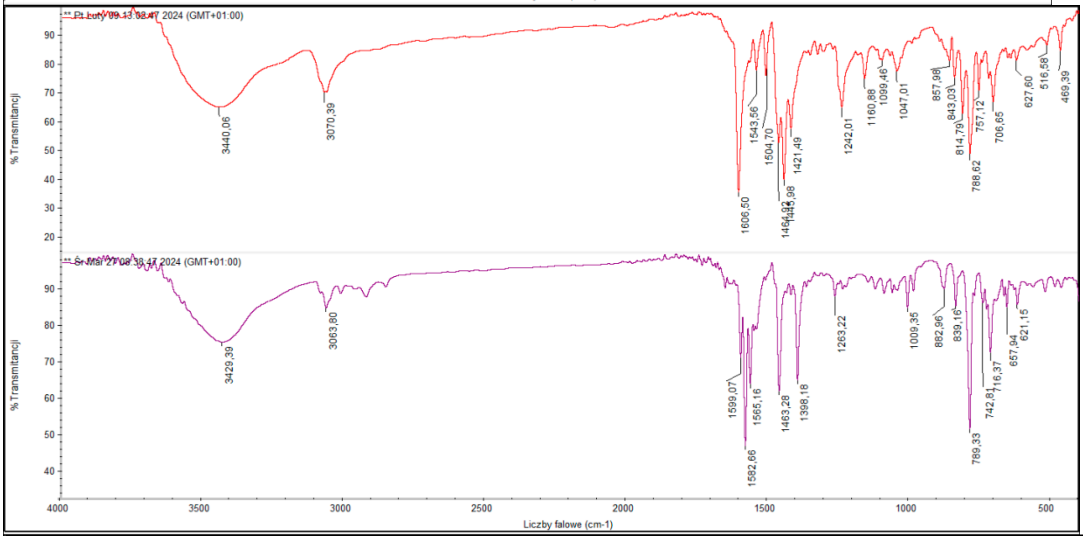

3

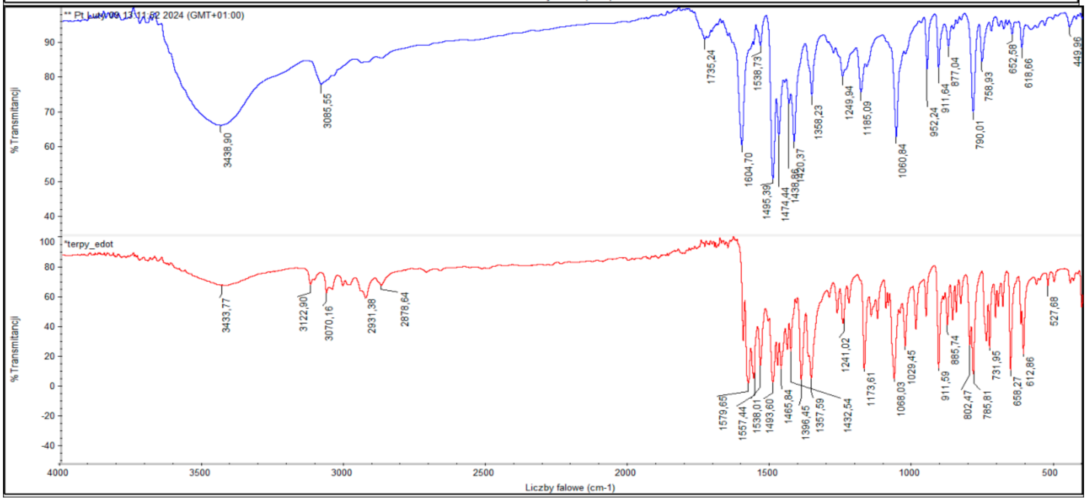

4

5

6

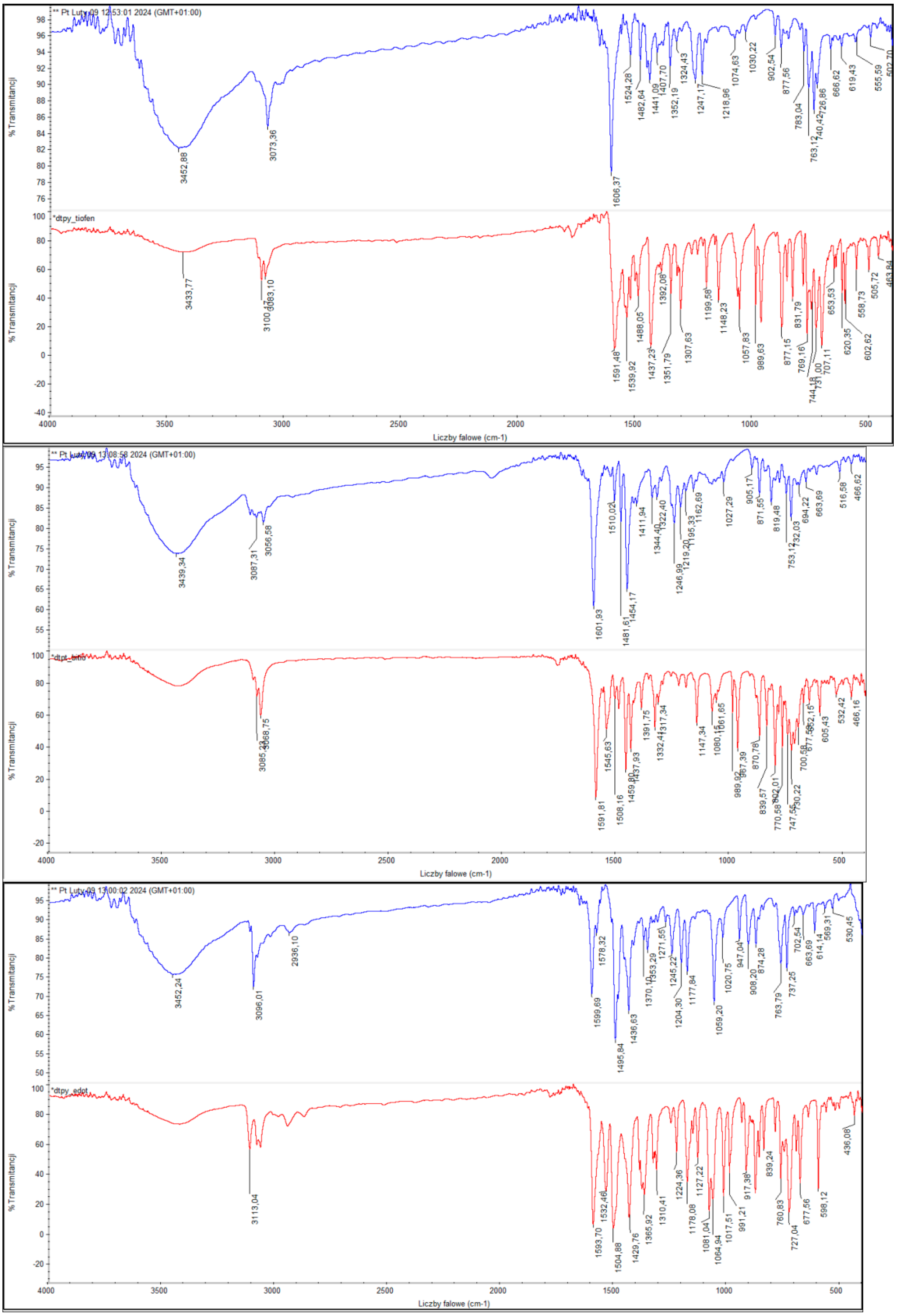

Figure S1. FT-IR spectra of **1-6**.

(a) Ir(III) complexes

1

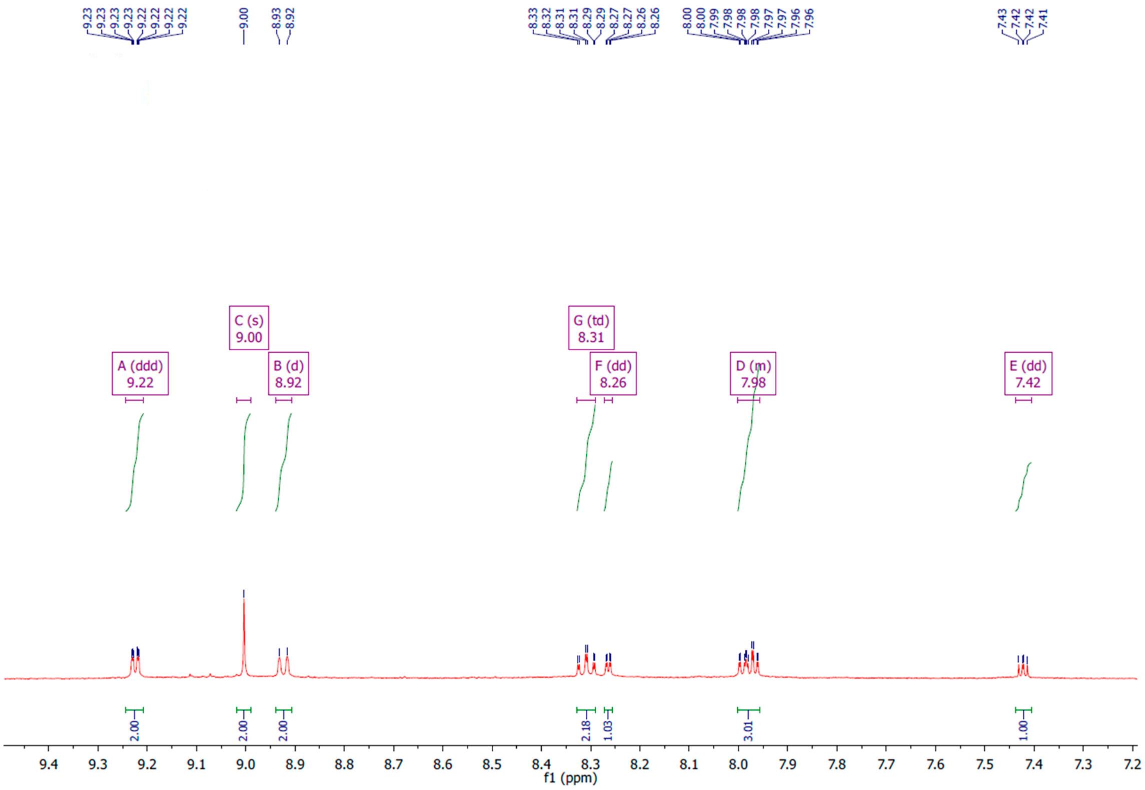

2

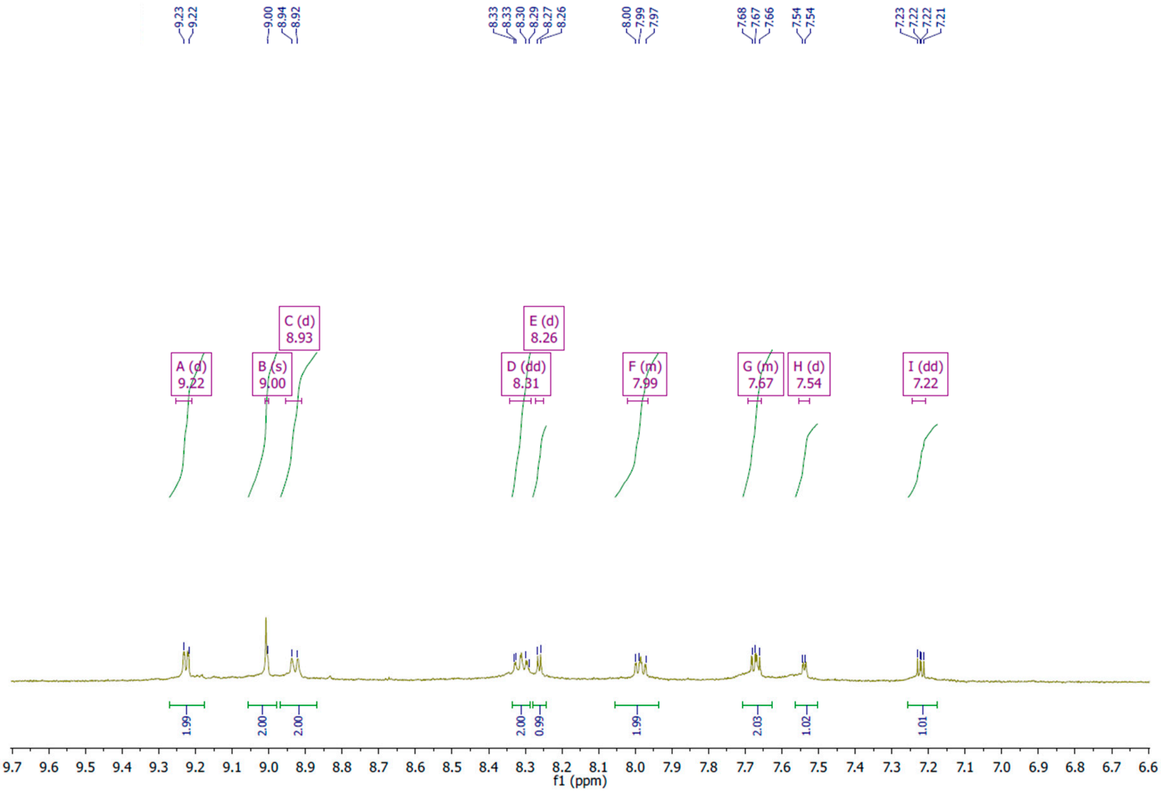

3

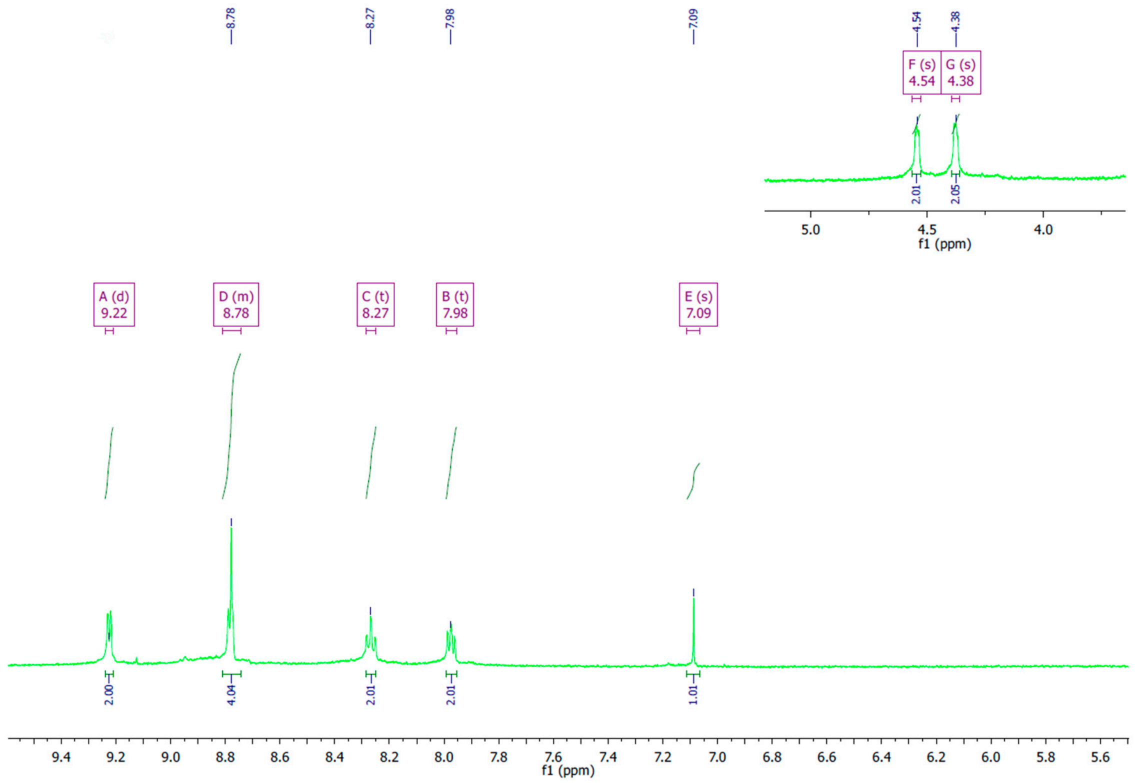

8.78
8.27
7.98
7.09
4.54
4.38
F (s) 4.54
G (s) 4.38
2.01
2.05
5.0
4.5
4.0
f1 (ppm)
A (d) 9.22
D (m) 8.78
C (t) 8.27
B (t) 7.98
E (s) 7.09
2.00
4.04
2.01
2.01
1.01
9.4 9.2 9.0 8.8 8.6 8.4 8.2 8.0 7.8 7.6 7.4 7.2 7.0 6.8 6.6 6.4 6.2 6.0 5.8 5.6
f1 (ppm)

4

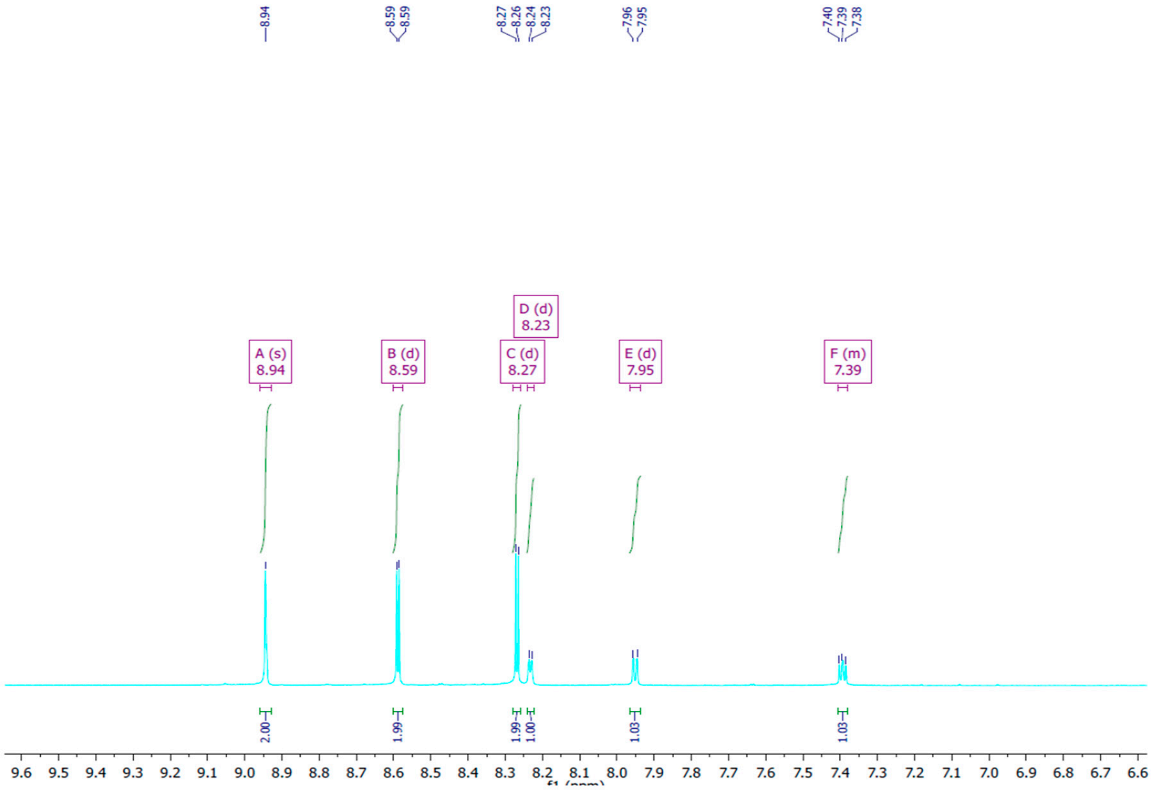

8.94
8.59
8.59
8.27
8.26
8.24
8.23
7.96
7.95
7.40
7.39
7.38
A (s) 8.94
B (d) 8.59
C (d) 8.27
D (d) 8.23
E (d) 7.95
F (m) 7.39
2.00
1.99
1.99
1.00
1.03
1.03
9.6 9.5 9.4 9.3 9.2 9.1 9.0 8.9 8.8 8.7 8.6 8.5 8.4 8.3 8.2 8.1 8.0 7.9 7.8 7.7 7.6 7.5 7.4 7.3 7.2 7.1 7.0 6.9 6.8 6.7 6.6

5

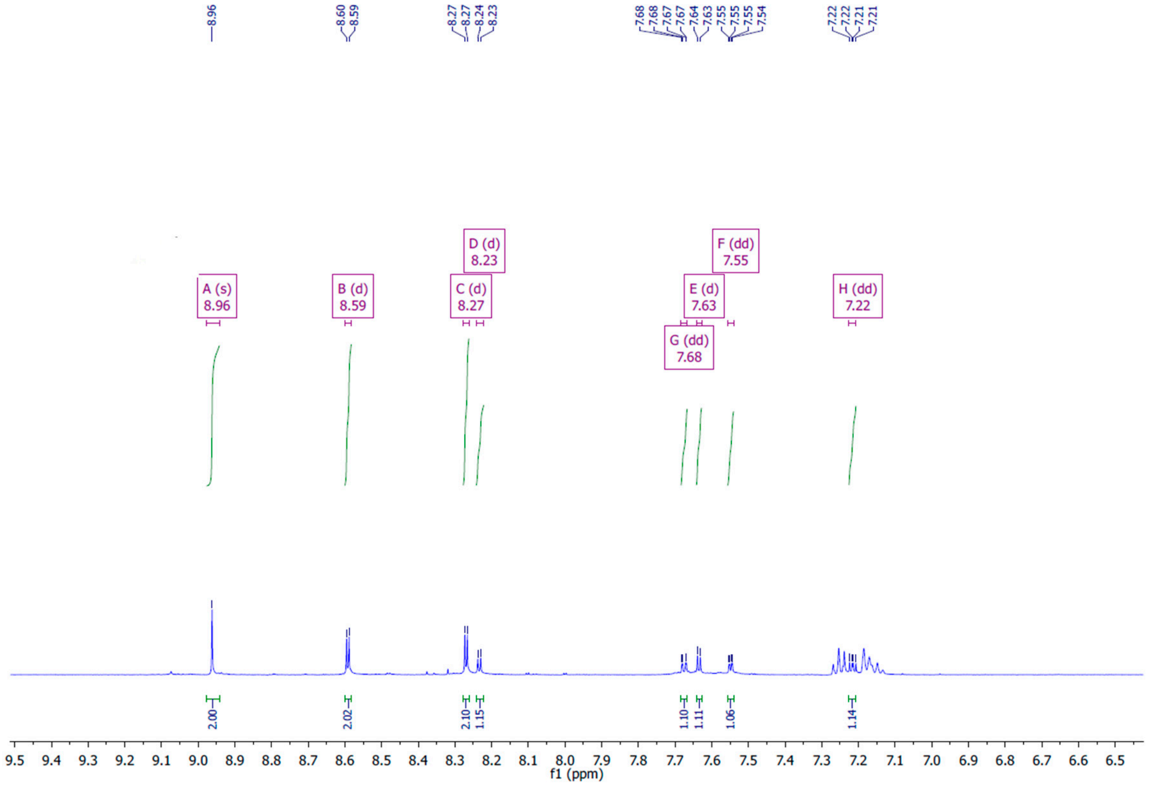

6

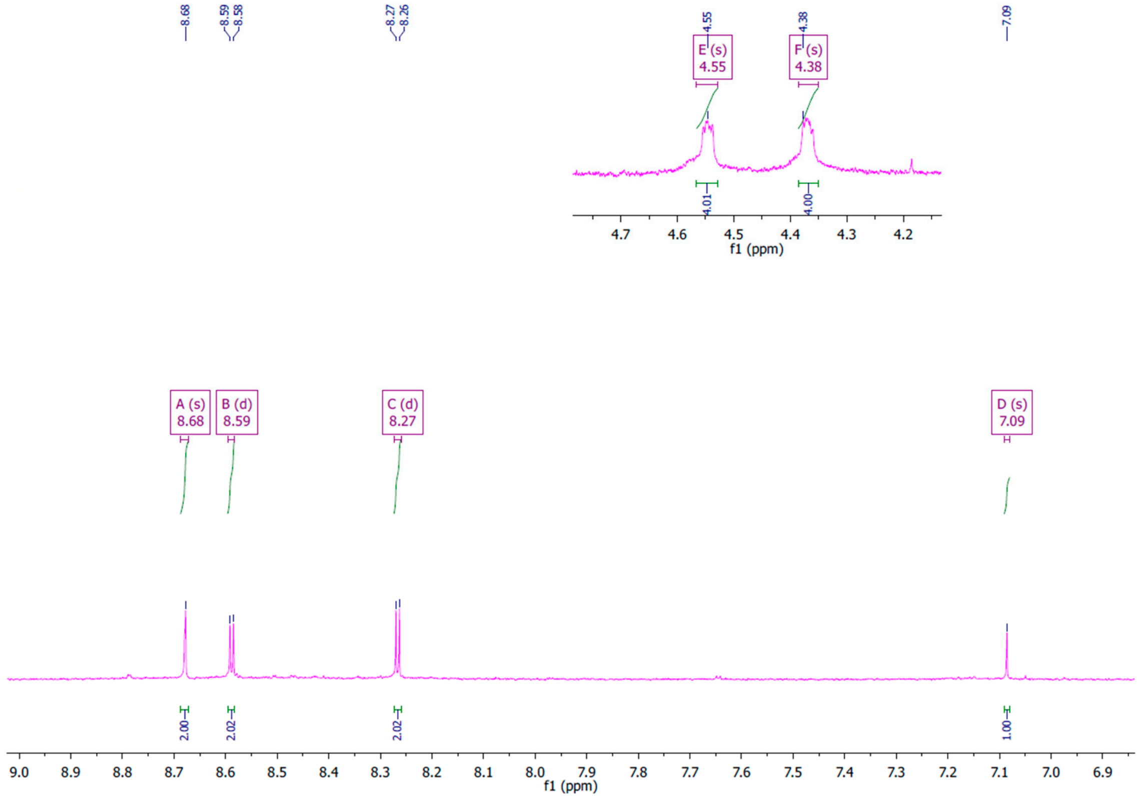

(b) ligand L^6

^{1}H NMR

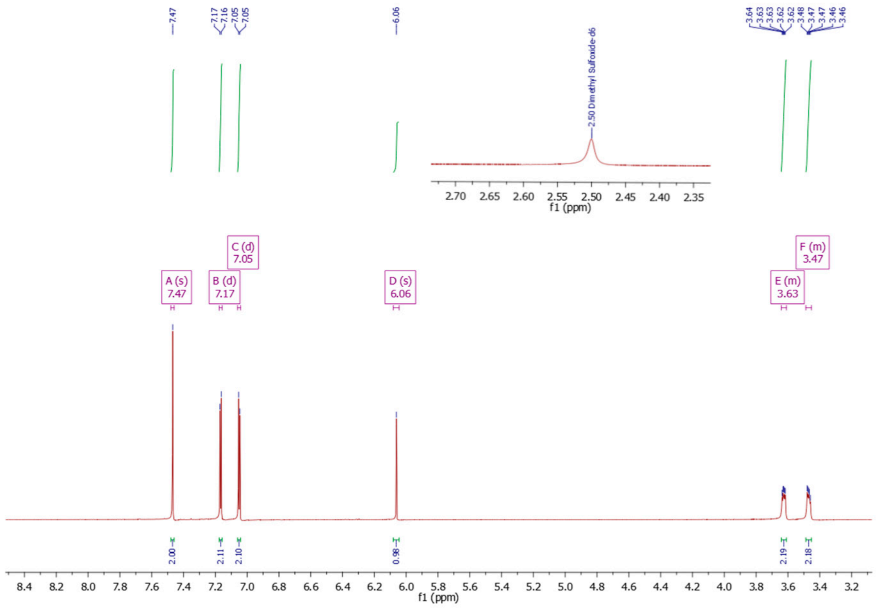

^{13}C NMR

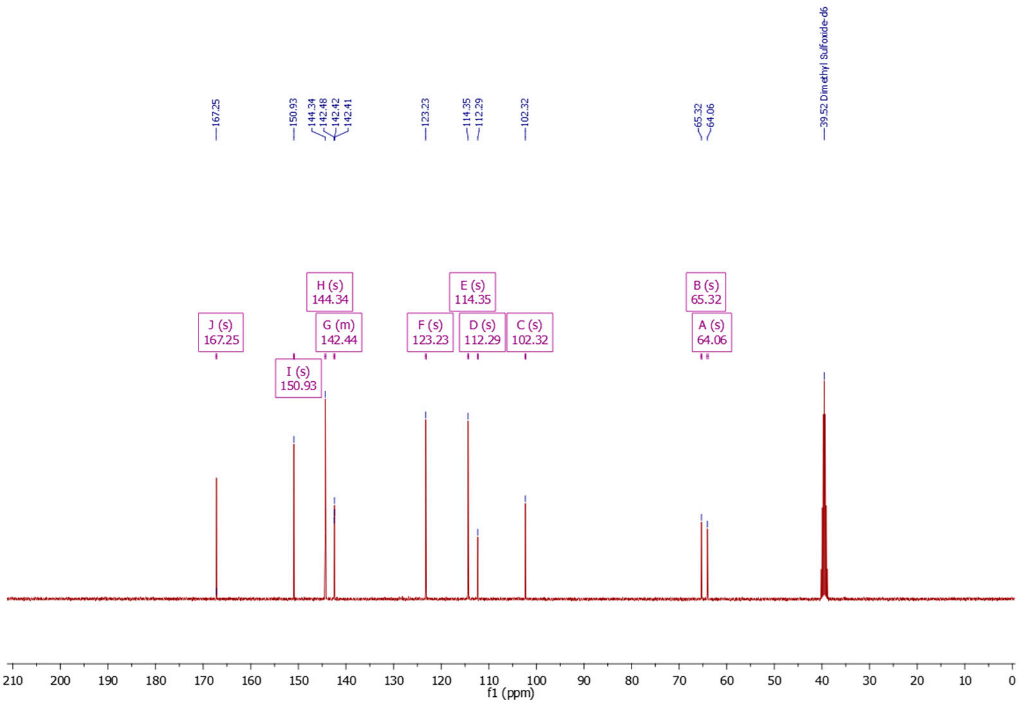

Figure S2. ^{1}H NMR spectra of **1-6 (a)** and ^{1}H NMR and ^{13}C NMR spectra of **L^{6} (b)**

X-Ray studies

Table S1. Crystal data and structure refinement.

	**1**	**6**
Empirical formula	$C_{19}H_{13}ClN_3SIr$	$C_{17}H_{11}Cl_3N_3O_2S_3Ir$
Formula weight	613.93	684.02
Temperature [K]	295.0(2)	295.0(2)
Wavelength [Å]	0.71073	0.71073
Crystal system	monoclinic	triclinic
Space group	$P2_1/c$	$P\bar{1}$

Unit cell dimensions [Å, °]	a = 10.4676(9)<br>b = 13.7381(8)<br>c = 13.4922(9)<br><br>β = 96.794(7)	a = 8.2819(4)<br>b = 10.5359(5)<br>c = 12.3457(5)<br>α = 86.616(4)<br>β = 84.771(4)<br>γ = 71.437(4)
Volume [Å ^{3}]	1926.6(2)	1016.44(8)
Z	4	2
Density (calculated) [Mg/m^3]	2.117	2.235
Absorption coefficient[mm^{-1}]	7.464	7.291
F(000)	1168	652
Crystal size [mm]	0.14 x 0.10 x 0.09	0.15 x 0.13 x 0.08
θ range for data collection [°]	3.38 to 25.05	3.49 to 25.05
Index ranges	$-14 \le h \le 13$<br>$-16 \le k \le 18$<br>$-18 \le l \le 13$	$-11 \le h \le 11$<br>$-14 \le k \le 14$<br>$-16 \le l \le 16$
Reflections collected	10384	9160
Independent reflections	4579 (R_{int}=0.0466)	4789 (R_{int}= 0.0453)
Completeness to 2θ [%]	99.7	99.7
Max. and min. transmission	1.00 and 0.492	1.00 and 0.257
Data / restraints / parameters	4579/ 0 / 244	4789/0 / 262
Goodness-of-fit on F^2	1.045	1.018
Final R indices [$I > 2\sigma(I)$]	*R1* = 0.0380<br>*wR2* = 0.0685	*R1* = 0.0431<br>*wR2* =0.0843
R indices (all data)	*R1* = 0.0607<br>*wR2* = 0.0797	*R1* = 0.0568<br>*wR2* = 0.0927
Largest diff. peak and hole[eÅ $^{-3}$]	1.059 and -0.924	1.784 and -1.224

Table S2. Selected bond lengths (Å) and angles (deg) for **1** and **6**.

**Bond lengths [Å]**			**Bond angles [°]**		
			**1**		
	experimental	calculated		experimental	calculated
Ir(1)–N(1)	2.044(4)	2.06763	N(2)–Ir(1)–N(1)	80.30(17)	80.506
Ir(1)–N(2)	1.939(4)	1.95812	N(2)–Ir(1)–N(3)	80.89(17)	80.512
Ir(1)–N(3)	2.040(4)	2.06728	N(3)–Ir(1)–N(1)	161.00(17)	161.018
Ir(1)–Cl(1)	2.3648(18)	2.46970	N(1)–Ir(1)–Cl(1)	91.95(15)	89.857
Ir(1)–Cl(2)	2.3816(14)	2.49149	N(1)–Ir(1)–Cl(2)	99.07(13)	99.718
Ir(1)–Cl(3)	2.3489(18)	2.46974	N(1)–Ir(1)–Cl(3)	88.54(15)	89.828
			N(2)–Ir(1)–Cl(1)	86.39(15)	89.868
			N(2)–Ir(1)–Cl(2)	177.30(16)	179.715
			N(2)–Ir(1)–Cl(3)	92.64(15)	89.539
			N(3)–Ir(1)–Cl(1)	89.49(15)	90.079
			N(3)–Ir(1)–Cl(2)	99.85(13)	99.265
			N(3)–Ir(1)–Cl(3)	89.69(15)	90.039
			Cl(1)–Ir(1)–Cl(2)	91.02(6)	90.310
			Cl(1)–Ir(1)–Cl(3)	178.82(5)	179.367
			Cl(2)–Ir(1)–Cl(3)	89.96(6)	90.284
			**6**		
Ir(1)–N(1)	2.032(5)	2.06593	N(2)–Ir(1)–N(1)	80.8(2)	79.614
Ir(1)–N(2)	1.960(5)	1.97156	N(2)–Ir(1)–N(3)	79.7(2)	79.617
Ir(1)–N(3)	2.022(5)	2.06553	N(3)–Ir(1)–N(1)	160.4(2)	159.230

Ir(1)–Cl(1)	2.3490(16)	2.46866	N(1)–Ir(1)–Cl(1)	91.93(14)	89.899
Ir(1)–Cl(2)	2.3507(17)	2.47937	N(1)–Ir(1)–Cl(2)	98.84(15)	100.339
Ir(1)–Cl(3)	2.3529(17)	2.46897	N(1)–Ir(1)–Cl(3)	88.05(14)	90.086
			N(2)–Ir(1)–Cl(1)	88.31(15)	89.586
			N(2)–Ir(1)–Cl(2)	177.75(14)	179.781
			N(2)–Ir(1)–Cl(3)	89.05(15)	89.935
			N(3)–Ir(1)–Cl(1)	89.16(14)	90.052
			N(3)–Ir(1)–Cl(2)	100.75(15)	100.431
			N(3)–Ir(1)–Cl(3)	89.97(14)	89.791
			Cl(1)–Ir(1)–Cl(2)	89.49(7)	90.200
			Cl(1)–Ir(1)–Cl(3)	177.32(6)	179.516
			Cl(2)–Ir(1)–Cl(3)	93.15(7)	90.278

Table S3. Short intra–and intermolecular contacts

D—H•••A	D—H	H•••A	D•••A[Å]	D—H•••A[°]
		**1**		
C(3)–H(3)•••Cl(3)[a]	0.93	2.70	3.370(6)	130.00
C(4)–H(4)•••Cl(3)[b]	0.93	2.69	3.614(6)	176.00
C(7)–H(7)•••S(1)	0.93	2.75	3.125(6)	105.00
C(7)–H(7)•••Cl(3)[b]	0.93	2.74	3.654(6)	168.00
C(17)–H(17)•••Cl(1)	0.93	2.83	3.747(6)	171.00
		**6**		
C(5)–H(5)•••O(3)	0.93	2.35	2.931(8)	120.00
C(7)–H(7)•••S(3)	0.93	2.81	3.148(6)	103.00
C(11)–H(11)•••O(2)[d]	0.93	2.59	3.505(8)	170.00
C(14)–H(14A)•••S(3)[e]	0.97	2.78	3.528(8)	135.00
C(15)–H(15A)•••Cl(2)[f]	0.97	2.76	3.537(9)	138.00
C(17)–H(17)•••Cl(3)	0.93	2.77	3.663(8)	160.00

Symmetry codes: (a): *x*,1/2-*y*,1/2+*z*; (b): 1-*x*,1/2+*y*,3/2-*z*; (c): *x*,1/2-*y*,-1/2+*z*; (d): 1+*x*, *y*,-1+*z*; (e): -1+*x*, *y*, *z*; (f): -1+*x*, *y*, 1+*z*; (g): *x*, *y*,1+*z*

Table S4. Short π•••π stacking interactions

Cg(I)•••Cg(J)	Cg(I)•••Cg(J) [Å]	α [°]	β [°]	γ [°]	Cg(I)-Perp [Å]	Cg(J)-Perp [Å]
			**1**			
Cg(1)•••Cg(2)[h]	3.902(3)	12.4(3)	22.07	34.30	3.223(3)	3.616(2)
Cg(2)•••Cg(3)[i]	3.960(3)	3.9(3)	28.82	27.02	3.528(2)	-3.470(2)
			**6**			
Cg(4)•••Cg(5)[j]	3.922(4)	9.5(3)	34.50	26.69	3.504(3)	-3.232(2)
Cg(5)•••Cg(6)[k]	3.953(3)	5.5(3)	28.30	25.92	-3.555(2)	-3.480(2)

*α = dihedral angle between *Cg*(I) and *Cg*(J); *Cg*(I)-Perp = Perpendicular distance of *Cg*(I) on ring J; *Cg*(J)-Perp = perpendicular distance of *Cg*(J) on ring I; β = angle *Cg*(I)→*Cg*(J) vector and normal to ring I; γ = angle *Cg*(I) →*Cg*(J) vector and normal to plane J;

[&]Symmetry code: (h) = 1-*x*,-1-*y*,-.*z;* (i): *x*,1/2-*y*,1/2+*z;* (j):-1+*x*, *y*, *z;* (k): 1-*x*,-*y*,1-*z*

Cg(1) is the centroid of the S(1)/C(16)/C(17)/C(18)/C(19)

Cg(2) is the centroid of the N(1)/C(1)/C(2)/C(3)/C(4)/C(5)

Cg(3) is the centroid of the N(2)/(6)/C(7)/C(8)/C(9)/C(10)

Cg(4) is the centroid of the S(1)/C(2)/C(1)/N(1)/C(3)

Cg(5) is the centroid of the S(2)/C(9)/N(3)/C(11)/C(10)

Cg(6) is the centroid of the N(2)/C(4)/C(5)/C(6)/C(7)/C(8)

Thermal properties

Table S5. TGA data for **1–6**

Compound	$T_{5\%}$	$T_{10\%}$	T_{max}
**1**	365	479	508
**2**	398	485	493
**3**	236	341	423
**4**	409	452	450
**5**	339	441	466
**6**	367	425	438

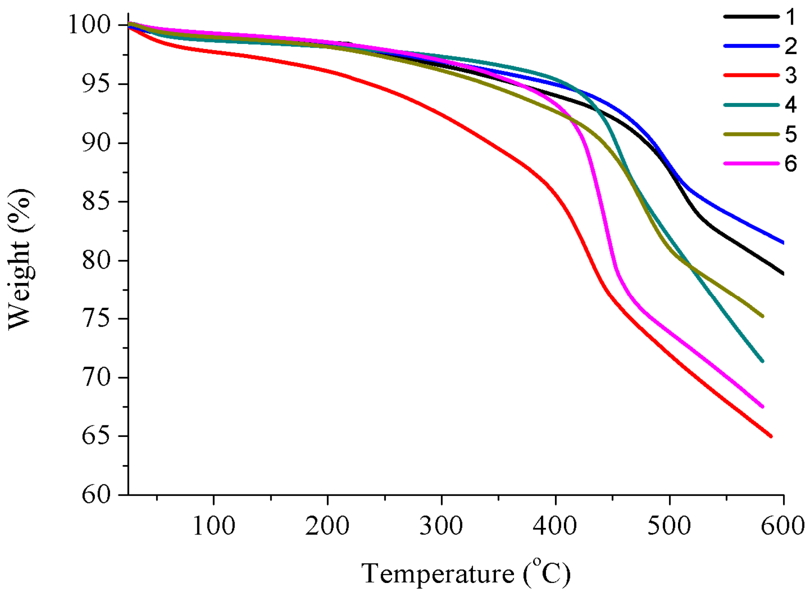

Figure S3. TGA of **1-6** under dry N_2 atmosphere

Spectroscopic studies

Table S6. The absorption maxima for complexes $[IrCl_3(L^1–L^6)]$ (**1-6**)

Compound	medium	λ/nm
**1**	DMSO	508, 409, 330, 280
**2**	DMSO	530, 497, 444, 394, 333, 288
**3**	DMSO	498, 410, 330, 288
**4**	DMSO	550, 426 , 350, 310

**5**	DMSO	563, 449, 348, 281
**6**	DMSO	535, 430, 343, 311

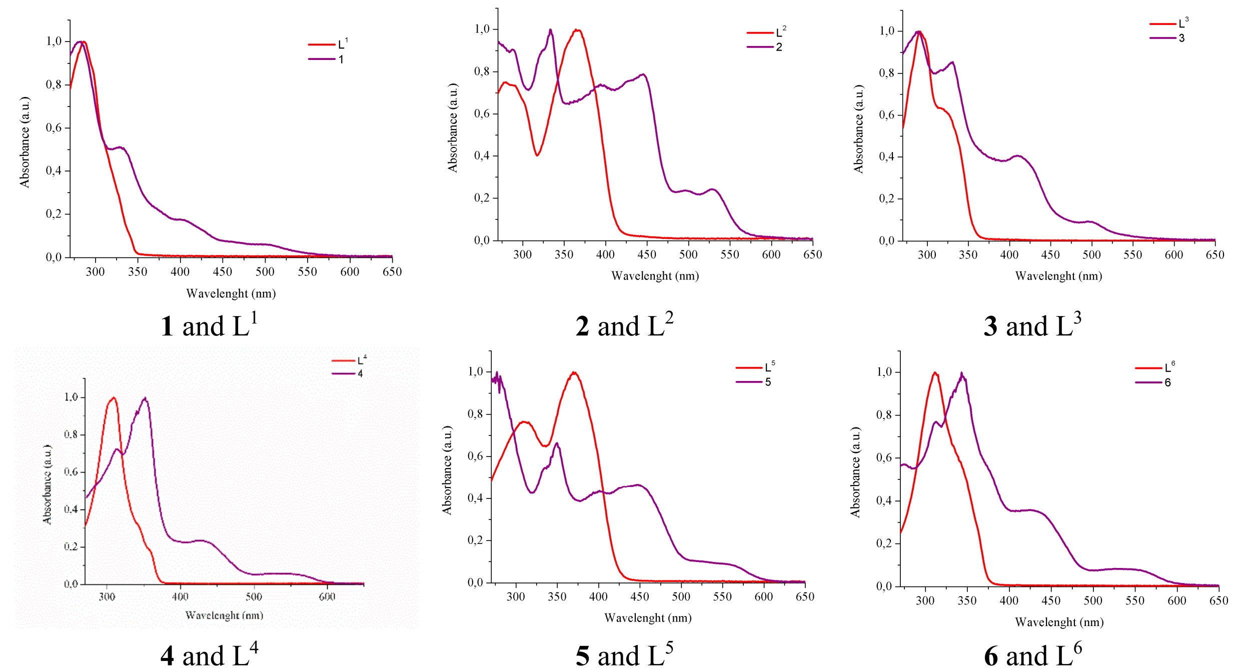

Figure S4. UV-Vis spectra of complexes **1-6** together with ligand L^1–L^6

Table S7. The energies and characters of the selected spin-allowed electronic transitions for **1** calculated with the TDDFT/PBE1PBE method, together with assignment to the experimental absorption bands.

Experimental Absorption	Major contribution	Character	Energy (eV)	Wavelength (nm)	Osc. Strength
	H-1→LUMO (97%)	MLCT	2.24	457.71	*0.0089*
508 nm	HOMO→LUMO (91%)	ILCT/ MLCT	2.27	451.43	*0.1552*
409 nm	HOMO→L+1 (95%)	ILCT/ MLCT	2.50	409.23	*0.0186*
	H-1→L+1 (95%)	MLCT	2.70	379.04	*0.0781*
	H-3→LUMO (89%)	ILCT	2.84	360.50	*0.2537*
	H-4→LUMO (92%)	LLCT	2.96	345.95	*0.0238*
330 nm	H-3→L+1 (67%)	ILCT	3.05	335.57	*0.0692*
	H-5→LUMO (57%)	ILCT	3.15	324.80	*0.063*
	HOMO→L+3 (91%)	ILCT	3.19	320.71	*0.1605*
	H-6→LUMO (65%)	ILCT	3.26	314.38	*0.0409*
	H-1→L+3 (33%), HOMO→L+4 (57%)	ILCT/MLCT	3.34	306.52	*0.0457*
280 nm	H-1→L+3 (50%), HOMO→L+4 (35%)	ILCT/MLCT	3.36	305.15	*0.0979*
	H-5→L+1 (59%)	ILCT	3.50	292.97	*0.0793*
	HOMO→L+5 (52%)	LLCT/ILCT	3.54	289.32	*0.0995*
	H-9→LUMO (81%)	ILCT/LLCT	3.59	285.34	*0.0934*
	H-6→L+1 (52%)	ILCT	3.66	279.58	*0.2937*

Table S8. The energies and characters of the selected spin-allowed electronic transitions for **2** calculated with the TDDFT/PBE1PBE method, together with assignment to the experimental absorption bands.

Experimental Absorption (nm)	Major contribution	Character	Energy (eV)	Wavelength (nm)	Osc. Strength
	HOMO→LUMO (94%)	ILCT	2.10	488.68	*0.6755*
530 nm	H-1→LUMO (96%)	MLCT/ILCT	2.22	461.16	*0.009*
497nm	H-2→L+3 (54%)<br>HOMO→L+3 (42%)	MLCT/ILCT	2.29	447.19	*0.0006*
	H-2→LUMO (83%)	MLCT/ILCT	2.43	422.23	*0.1199*
	H-1→L+1 (92%)	MLCT	2.71	378.07	*0.0505*
	H-2→L+1 (61%)	MLCT	2.72	375.96	*0.0174*
444 nm<br>394 nm	H-3→L+3 (65%)<br>H-2→L+1 (30%)	ILCT	2.77	369.10	*0.0047*
	HOMO→L+2 (95%)	ILCT	2.83	361.56	*0.4289*
	H-4→LUMO (97%)	LLCT	2.92	350.34	*0.0087*
	H-6→LUMO (71%)	ILCT	3.08	331.75	*0.1075*
	H-1→L+2 (64%)	MLCT	3.21	319.37	*0.0633*
333 nm	H-2→L+2 (56%)<br>HOMO→L+5 (29%)	ILCT/MLCT	3.24	315.71	*0.061*
	H-4→L+1 (71%)	ILCT	3.29	310.99	*0.0246*
288 nm	H-2→L+5 (32%),<br>HOMO→L+6 (17%)	ILCT	3.67	278.63	*0.0151*
	H-7→L+1 (44%)<br>H-6→L+1 (29%)	ILCT	3.71	275.91	*0.1285*

Table S9. The energies and characters of the selected spin-allowed electronic transitions for **3** calculated with the TDDFT/PBE1PBE method, together with assignment to the experimental absorption bands.

Experimental Absorption (nm)	Major contribution	Character	Energy (eV)	Wavelength (nm)	Osc. Strength
498 nm	HOMO→LUMO (89%)	ILCT/MLCT	2.24	456.18	*0.2336*
	H-1→LUMO (96%)	MLCT	2.27	451.76	*0.0123*
	HOMO→L+1 (94%)	ILCT	2.47	414.82	*0.0144*
	H-2→LUMO (90%)	ILCT	2.63	388.80	*0.0575*
410 nm	H-4→LUMO (34%)<br>H-1→L+1 (52%)	LLCT/MLCT	2.72	376.82	*0.2043*
	H-4→LUMO (44%)<br>H-1→L+1 (29%)	MLCT/ILCT	2.76	371.56	*0.0298*
	H-2→L+1 (93%)	ILCT	2.93	349.12	*0.0125*
	H-4→L+1 (91%)	MLCT/ILCT	2.97	344.97	*0.0192*
330 nm	HOMO→L+3 (91%)	ILCT	3.14	326.27	*0.2529*
	H-6→LUMO (84%)	ILCT	3.15	324.66	*0.1552*
	H-1→L+3 (80%)	MLCT/ILCT	3.36	305.03	*0.1286*
	HOMO→L+5 (82%)	ILCT	3.47	295.17	*0.1845*
288 nm	H-5→L+2 (68%)	LLCT	3.47	295.02	*0.0112*
	H-3→L+6 (86%)	ILCT	3.54	289.46	*0.0319*
	H-6→L+1 (35%),	ILCT/LLC	3.64	281.56	*0.2757*

	H-4→L+3 (42%),	T

Table S10. The energies and characters of the selected spin-allowed electronic transitions for **4** calculated with the TDDFT/PBE1PBE method, together with assignment to the experimental absorption bands.

Experimental Absorption (nm)	Major contribution	Character	Energy (eV)	Wavelength (nm)	Osc. Strength
	H-1→LUMO (98%)	MLCT	2.01	509.85	*0.0004*
550 nm	HOMO→LUMO (87%)	MLCT/ILCT	2.09	490.87	*0.1256*
	HOMO→L+1 (95%)	MLCT	2.28	448.21	*0.0206*
	H-1→L+1 (90%)	MLCT	2.50	409.42	*0.1508*
426 nm	H-3→LUMO (82%)	ILCT/LLCT	2.63	388.82	*0.1838*
	H-4→LUMO (64%)	LLCT	2.74	373.84	*0.0158*
350 nm	H-5→LUMO (44%), H-3→L+1 (50%)	ILCT	2.81	364.05	*0.1145*
	H-5→LUMO (49%), H-3→L+1 (43%)	ILCT	2.89	354.89	*0.0829*
	H-6→LUMO (74%)	ILCT	3.05	335.62	*0.0254*
	H-5→L+1 (55%), HOMO→L+3 (31%)	ILCT	3.26	314.42	*0.4429*
310 nm	H-10→LUMO (92%)	LLCT/ILCT	3.45	297.06	*0.1483*
	H-11→LUMO (78%)	LLCT/ILCT	3.64	281.58	*0.1068*
	H-9→L+1 (96%)	ILCT	3.66	279.89	*0.2507*
	H-7→L+2 (92%)	ILCT	3.71	275.67	*0.0281*
	H-3→L+3 (71%)	LLCT/ILCT	3.78	270.73	*0.0262*

Table S11. The energies and characters of the selected spin-allowed electronic transitions for **5** calculated with the TDDFT/PBE1PBE method, together with assignment to the experimental absorption bands.

Experimental Absorption (nm)	Major contribution	Character	Energy (eV)	Wavelength (nm)	Osc. Strength
563 nm	HOMO→LUMO (93%)	ILCT	1.92	534.51	0.5582
	H-1→LUMO (97%)	MLCT	2.00	511.13	0.0028
	HOMO→L+1 (93%)	ILCT	2.11	485.13	0.0104
449 nm	H-2→LUMO (82%)	ILCT/LLCT/MLCT	2.24	456.51	0.1082
	H-2→L+1 (74%)	ILCT/LLCT/MLCT	2.50	409.16	0.0486
	H-1→L+1 (68%)	MLCT/ILCT	2.51	407.57	0.0611
	HOMO→L+2 (96%)	ILCT	2.75	372.18	0.6032
	H-6→LUMO (76%)	ILCT	2.84	360.91	0.1875
348 nm	H-5→LUMO (76%)	ILCT	2.97	344.99	0.0165
	H-7→LUMO (91%)	ILCT	3.10	330.08	0.0201
	H-2→L+2 (64%)	ILCT/LLCT	3.21	319.02	0.0717
281 nm	H-9→LUMO (35%) H-5→L+1 (40%)	ILCT	3.29	311.53	0.0481

	H-6→L+1 (65%)	ILCT	3.34	306.42	0.0814
	H-12→LUMO (88%)	ILCT	3.43	298.53	0.0899
	H-9→L+1 (79%)	ILCT	3.55	288.58	0.112
	H-11→L+1 (87%)	ILCT	3.69	277.84	0.141
	H-8→L+3 (64%)	ILCT	3.72	275.25	0.0204

Table S12. The energies and characters of the selected spin-allowed electronic transitions for **6** calculated with the TDDFT/PBE1PBE method, together with assignment to the experimental absorption bands.

Experimental Absorption (nm)	Major contribution	Character	Energy (eV)	Wavelength (nm)	Osc. Strength
535 nm	H-1→LUMO (95%)	MLCT	2.04	502.88	*0.0009*
	HOMO→LUMO (86%)	ILCT/MLCT	2.07	495.72	*0.1909*
	HOMO→L+1 (94%)	ILCT	2.25	455.79	*0.0164*
430 nm	H-2→LUMO (88%)	ILCT	2.42	423.14	*0.0434*
	H-4→LUMO (32%)<br>H-1→L+1 (49%)	ILCT/MLCT	2.50	409.74	*0.245*
	H-2→L+1 (94%)	ILCT	2.67	383.95	*0.0099*
343 nm	H-4→L+1 (61%)<br>H-3→L+2 (32%)	ILCT/LLCT	2.71	377.26	*0.0144*
	H-6→LUMO (94%)	ILCT/LLCT	2.89	354.81	*0.214*
	HOMO→L+3 (95%)	ILCT	3.19	320.53	*0.4757*
	H-6→L+1 (89%)	ILCT/LLCT	3.32	308.50	*0.1588*
	H-10→LUMO (41%)<br>H-9→LUMO (49%)	ILCT/LLCT	3.48	294.37	*0.0741*
	H-2→L+3 (89%)	ILCT	3.53	290.15	*0.107*
311 nm	H-11→LUMO (68%)<br>H-4→L+3 (26%)	ILCT/LLCT	3.64	281.54	*0.1277*
	H-9→L+1 (94%)	ILCT	3.68	278.14	*0.268*
	H-10→L+1 (89%)	ILCT/LLCT	3.79	270.08	*0.0193*

1

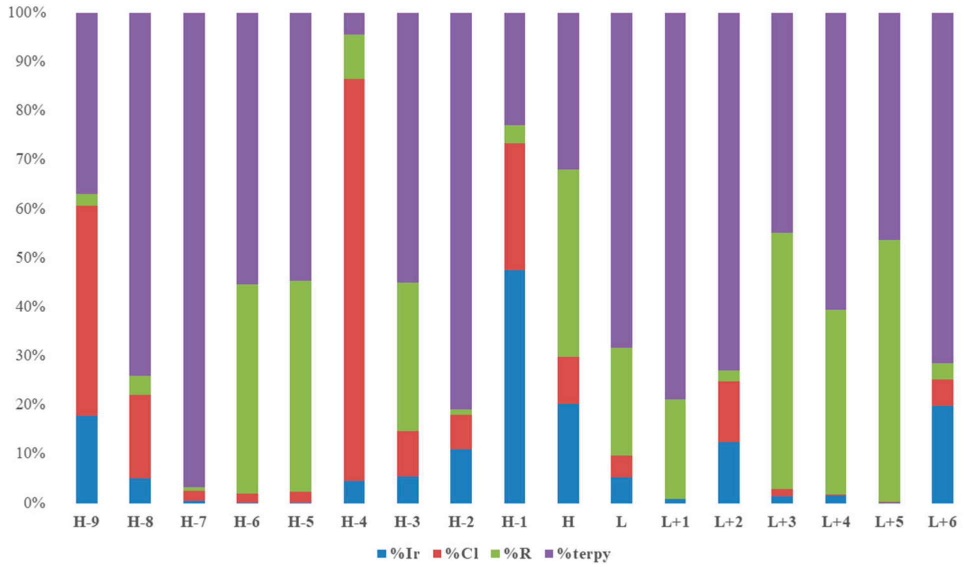
100%
90%
80%
70%
60%
50%
40%
30%
20%
10%
0%
H-9 H-8 H-7 H-6 H-5 H-4 H-3 H-2 H-1 H L L+1 L+2 L+3 L+4 L+5 L+6
%Ir %Cl %R %terpy

2

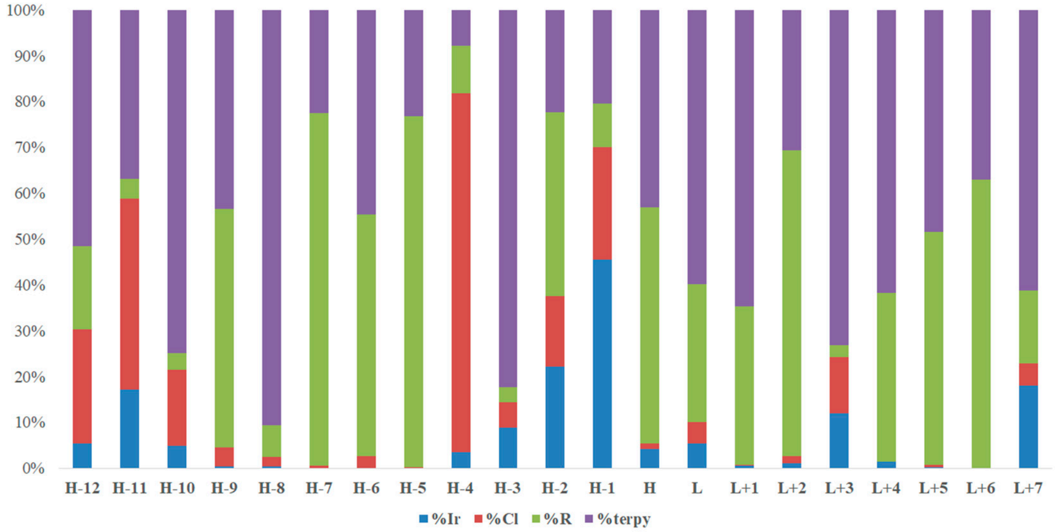
100%
90%
80%
70%
60%
50%
40%
30%
20%
10%
0%
H-12 H-11 H-10 H-9 H-8 H-7 H-6 H-5 H-4 H-3 H-2 H-1 H L L+1 L+2 L+3 L+4 L+5 L+6 L+7
%Ir %Cl %R %terpy

3

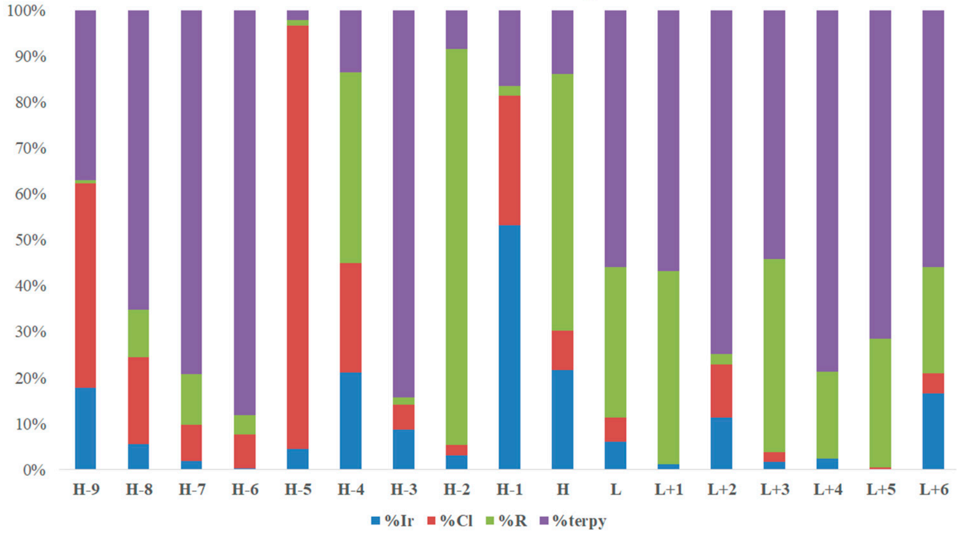
100%
90%
80%
70%
60%
50%
40%
30%
20%
10%
0%
H-9 H-8 H-7 H-6 H-5 H-4 H-3 H-2 H-1 H L L+1 L+2 L+3 L+4 L+5 L+6
%Ir %Cl %R %terpy

4

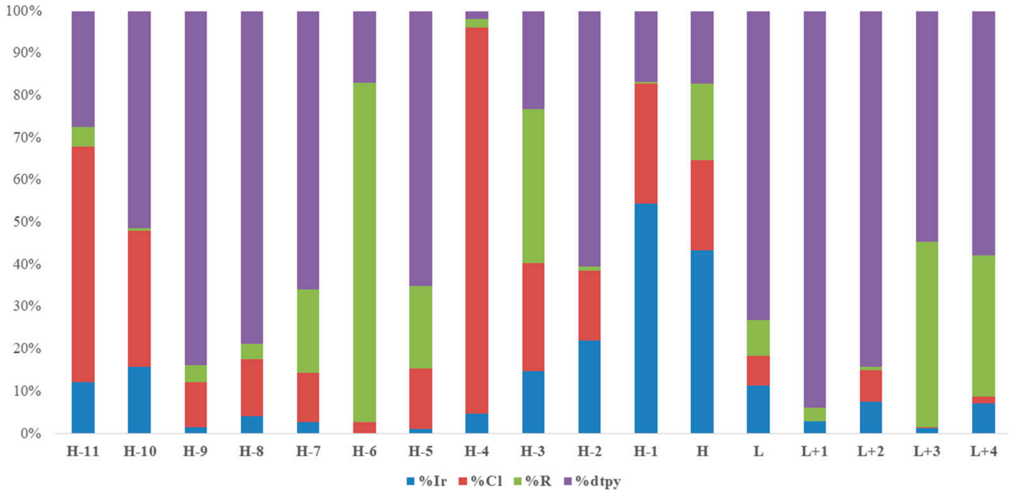

5

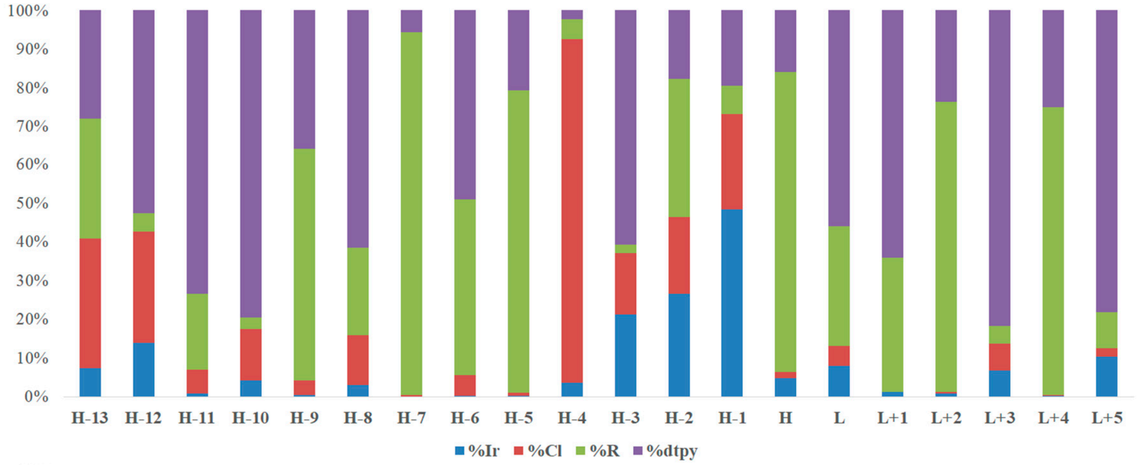

6

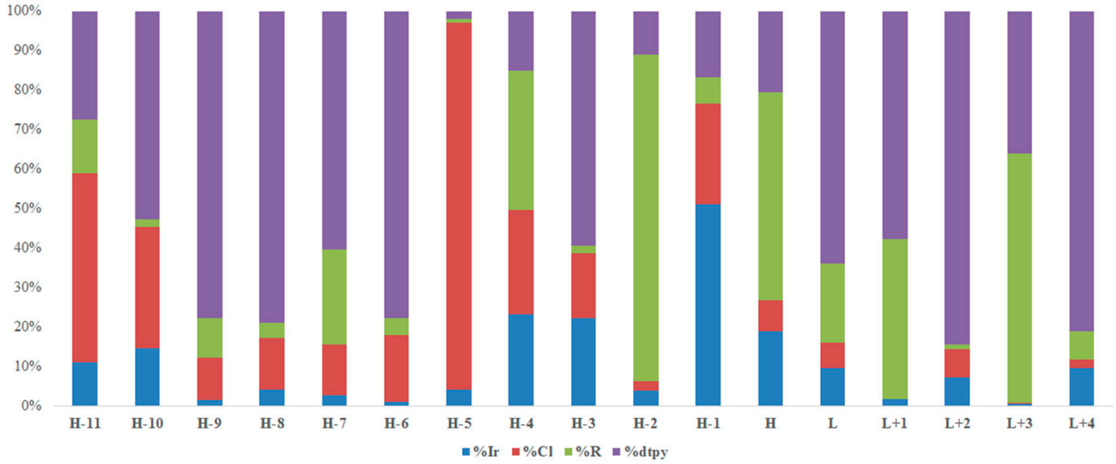

Figure S5. Composition of frontier molecular orbitals of complexes **1-6** (blue – Ir, red – 3Cl green – R’ substituent, violet – *terpy*/*dtpy* skeleton).

Table S13. Frontier molecular orbitals of complexes **1-6**

	H-3	H-2	H-1	H	L	L+1	L+2	L+3
**1**	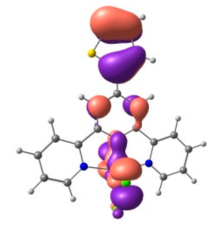	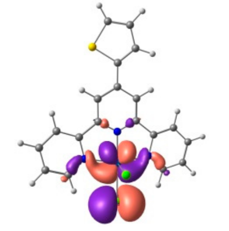	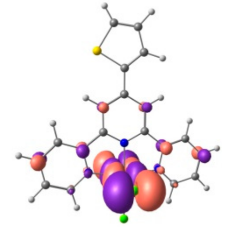	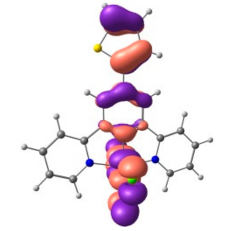	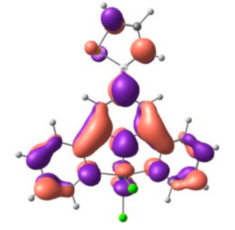	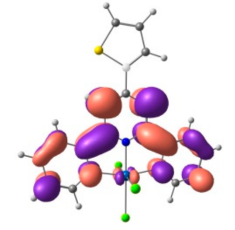	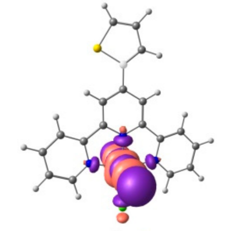	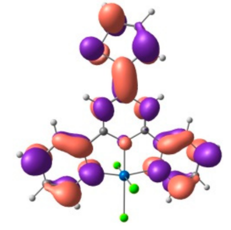
**2**	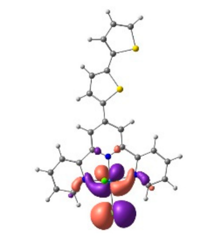	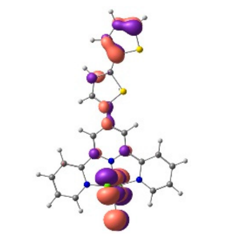	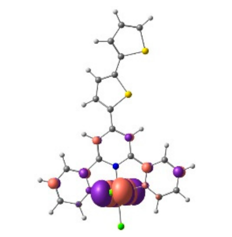	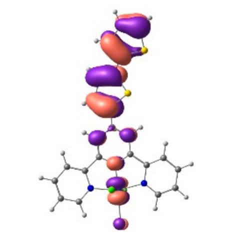	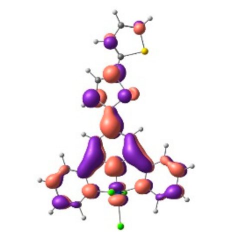	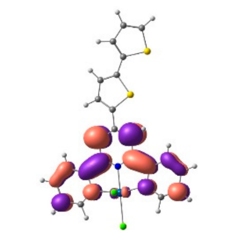	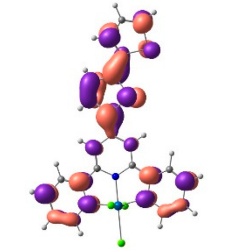	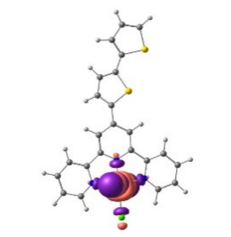
**3**	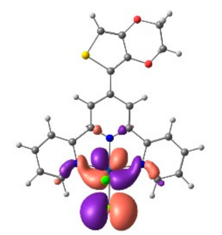	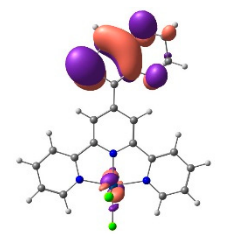	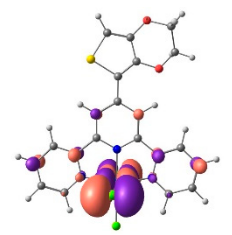	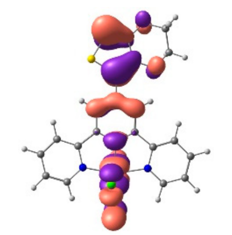	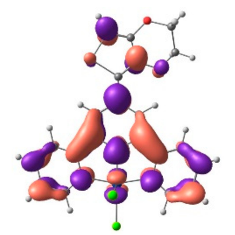	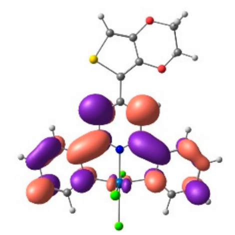	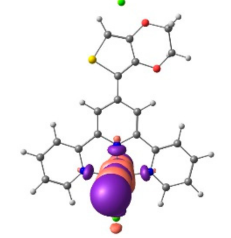	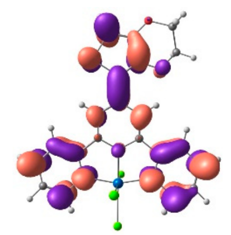
**4**	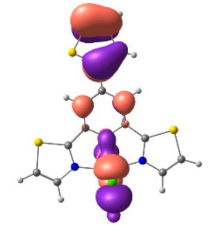	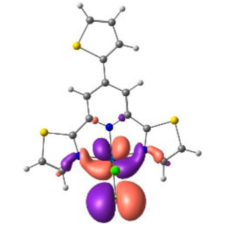	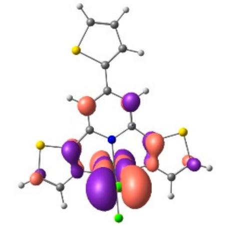	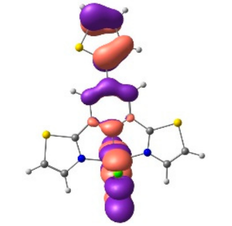	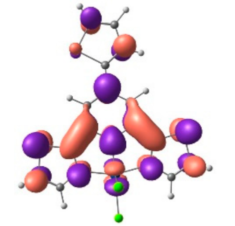	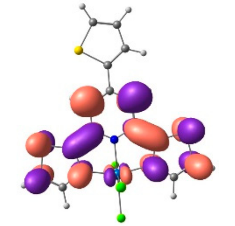	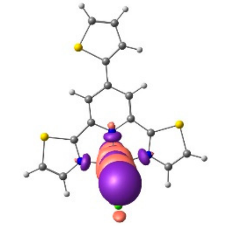	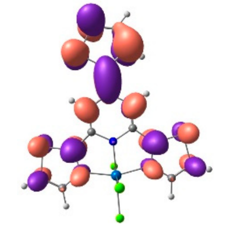

5

6

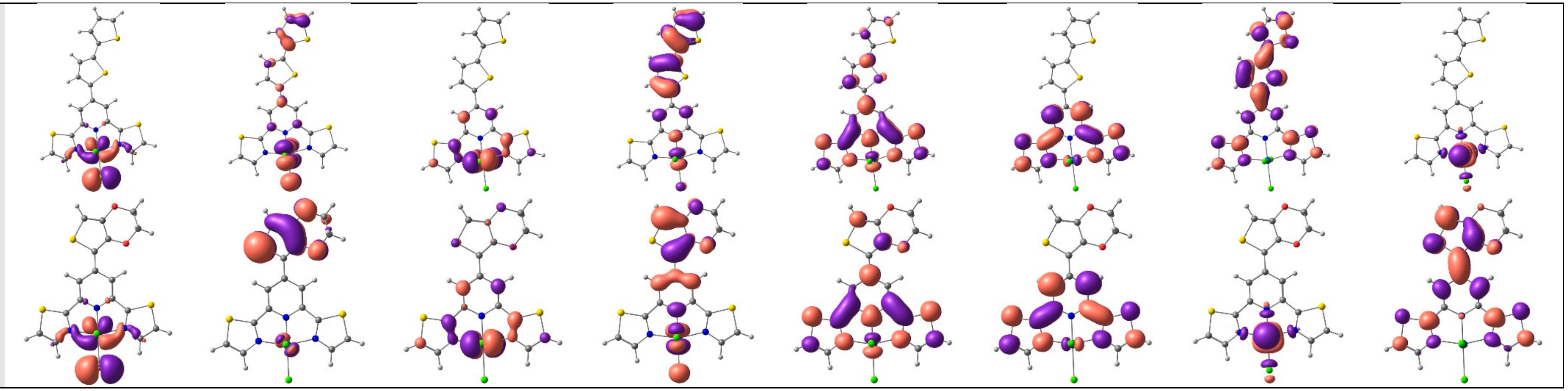

Electrochemistry

Table S14. Electrochemical properties of the Ir(III) complexes in DMF.

Compound	E_{oneset} [V]	E_{red} [V]
1	-1.49	-1.61
2	-1.43	-1.59
3	-1.50	-1.63
4	-1.39	-1.51
5	-1.29	-1.43
6	-1.42	-1.54

Table S15. Structural and photophysical comparison of related Ir(III) complexes.

Compound	Bond lenghts [Å]		Angles [°]		λ [nm]		λem	λex	τ [μs]	φ	Ref
	Ir-N	Ir–Cl	Cl–Ir–Cl	N–Ir–Cl	π-π*	MCLT					
[IrCl$_3$(terpy)]			–		266 282, 313, 327	408, 523	584	327	0.29	0.075	1
[IrCl$_3$(Clterpy)			–		265 283 312 327	413 527	600	327	0.43	0.094	1
[IrCl$_3$(Bterpy)]			–		269 283 310 324	405 514	591	324	0.49	0.0069	1
[IrCl$_3$(Brterpy)]			–		293 327 340	419	567	327	0.5	0.019	1
[IrCl$_3$(tterpy)]			–		328 348 373	521	591	328	0.66	0.0071	1
[IrCl$_3$T$_0$]			–		no data	465 533	–	–	–	–	
[IrCl$_3$T$_1$]			–		no data	476 538	–	–	–	–	2
[IrCl$_3$(tppz)]	1.916<br>2.031<br>2.031	2.349<br>2.363<br>2.363	179.9	180.0	370 280 250 210	427 558	632	No data	1.31	–	3
[IrCl$_3$(3-MeO-Phtpy)]	2.046(4)<br>1.952(4)<br>2.049(4)	2.359(2)<br>2.365(2)<br>2.352(2)	177.97(6)	179.3(1)			–				4
[IrCl$_3$(2-MeO-Phtpy)]	2.060(7)<br>1.937(7)<br>2.038(6)	2.357(2)<br>2.376(2)<br>2.345(3)	177.72(9)	179.8(2)			–				4
[IrCl$_3$(4-MeO-Phtpy)]	2.042(4)<br>1.948(5)<br>2.039(4)	2.355(2)<br>2.343(2)<br>2.379(2)	179.87(6)	176.9(1)			–				4
[IrCl$_3$(ttepy)]·DMSO	2.037(4)<br>1.947(4)<br>2.041(4)	2.355(2)<br>2.386(1)<br>2.372(1)	178.90(5)	178.6(1)			–				5
[IrCl$_3$(terpy)]	2.044(3)<br>1.927(3)<br>2.049(3)	2.3556(7)<br>2.3466(8)<br>2.370(1)	179.51(3)	177.34(8)			–				6
[IrCl$_3$(bupzpy)]·2DMF	2.050(4)<br>1.980(5)<br>2.058(4)	2.364(2)<br>2.366(2)<br>2.357(2)	179.15(6)	175.9(2)			–				7
[IrCl$_3$(L$_3$HMe)]	Ir(1)<br>2.046(7)<br>2.017(5)<br>2.032(7)	Ir(1)<br>2.364(2)<br>2.383(2)<br>2.370(2)	Ir(1)<br>176.64(6)<br>Ir(2)<br>179.42(7)	Ir(1)<br>176.5(2)<br>Ir(2)<br>175.8(2)			–				8

	Ir(2)	Ir(2)				
	2.059(5)	2.368(2)				
	2.045(5)	2.361(2)				
	2.024(6)	2.338(2)				
	2.050(4)	2.376(2)				
[$IrCl_3$(ferterpy)]·2CH_3CN	1.939(5)	2.351(1)	178.55(5)	178.8(1)	–	9
	2.042(4)	2.358(1)				

Terpy = 2,2':6',2"-terpyridine, **Clterpy** = 4'-chloro-2,2':6',2"-terpyridine; **Bterpy** = 4,4',4"-tri-(tert-butyl)- 2,2':6',2"-terpyridine, **Brterpy** = 6,6'-dibromo–2,2':6',2"-terpyridine; **tterpy** = 4'-(4-tolyl)-2,2':6',2"-terpyridine; **tppz** = 2,3,5,6-tetrakis(2-pyridyl)pyrazine; **T_0**=4′-(Phenyl-p-dibutylamino)-2,2′:6′,2″-terpyridine; **T_1**= 4′-(4-{2-[4-(N,N-Dibutylamino)phenyl]ethenyl}phenyl)-2,2′:6′,2″-terpyridine; **bupzpy** = 2,6-bis(5-tert-butyl-1Hpyrazol-3-yl)pyridin; **L_3HMe** – tridendate ligand; **ferterpy** = 4'-ferrocenyl-2,2':6',2"-terpyridine

1. Yoshikawa N.; Yamabe S.; Kanehisa N.; Inoue T.; Takashima H.; Tsukahara K.; Detailed Description of the Metal-to-Ligand Charge-Transfer State in Monoterpyridine IrIII Complexes. *Eur. J. Inorg. Chem*. **2009**, 2067–2073.

2 Tessore F.; Roberto D.; Ugo R.; Pizzotti M. Terpyridine Zn(II), Ru(III), and Ir(III) Complexes: The Relevant Role of the Nature of the Metal Ion and of the Ancillary Ligands on the Second-Order Nonlinear Response of Terpyridines Carrying Electron Donor or Electron Acceptor Groups. *Inorg. Chem.* **2005**, *44*, 8967−8978.

3. Vogler L. M.; Scott B.; Brewer K. J., Investigation of the Photochemical, Electrochemical, and Spectroelectrochemical Properties of an Iridium(III)/Ruthenium(II) Mixed-Metal Complex Bridged by 2,3,5,6-Tetrakis(2-pyridyl)pyrazine. *Inorg. Chem*. **1993**, *32*, 898–903.

4. Qin, Q.P.; Meng, T.; Tan, M.X.; Liu, Y.C.; Luo, X.J.; Zou, B.Q.; Liang,H. Synthesis and in vitro biological evaluation of three 40 -(4- methoxyphenyl)-2,2′ :6′,2″-terpyridine iridium(III) complexes as new telomerase inhibitors. *Eur. J. Med. Chem*. **2018**, *143*, 1387–1395.

5. Hinkle, L. M.; Young Jr, V.G.; Mann, K.R. Mono- and bis-tolylterpyridine iridium(III) complexes. *Acta Cryst.,Sect. C: Crystal Structure Communications*, **2010**, *66*, m62.

6. Dobroschke,M.; Geldmacher,Y.; Ott, I.; Harlos, M.; Kater, L.; Wagner, L.; Gust, R.; Sheldrick, W.S.; Prokop, A. Cytotoxic Rhodium(III) and Iridium(III) Polypyridyl Complexes: Structure–Activity Relationships, Antileukemic Activity, and Apoptosis Induction. *ChemMedChem*, **2009**, *4*, 177–187.

7. Toda, T.; Saitoh, K.; Yoshinari, A.; Ikariya, T.; Kuwata,S. Synthesis and Structures of NCN Pincer-Type Ruthenium and Iridium Complexes Bearing Protic Pyrazole Arms. *Organometallics*, **2017**, *36*, 1188–1195.

8. Zheng, Z.; Zhu, Z.-L.; Ho, C.-L.; Yiu, S.-M.; Lee, C.-S. Songwut Suramitr, Supa Hannongbua, Yun Chi, Stepwise Access of Emissive Ir(III) Complexes Bearing a Multi Dentate Heteroaromatic Chelate: Fundamentals and Applications. *Inorg. Chem.* **2022**, *61*, 4384–4393.

9. Davaasuren, B.; Padhy, H.; Rothenberger, A. Crystal structure of trichlorido(4′-ferrocenyl-2,2′:6′,2″-terpyridine-κ^3N,N′,N″)iridium(III) acetonitrile disolvate. *Acta Cryst.Sec. E: Cryst. Commun.*, **2015**, *71*, m69.
